# scH16S‐Seq Maps *Klebsiella pneumoniae*‐Associated Myeloid States and Reveals a CD38–NAD Immunometabolic Axis That Impairs Lysosomal Acidification in Sepsis

**DOI:** 10.1002/advs.78127

**Published:** 2026-09-30

**Authors:** Yangguang Li, Yilong Yu, Fang Qin, Shenxing Li, Xuanheng Li, Peizhao Liu, Juanhan Liu, Haiqing Liu, Songqi Jing, Jianghao Zou, Sicheng Li, Jiayang Li, Tao Zheng, Yun Zhao, Jianan Ren, Xiuwen Wu

**Affiliations:** ^1^ Research Institute of General Surgery Jinling Hospital Affiliated Hospital of Medical School Nanjing University Nanjing People's Republic of China; ^2^ Department of General Surgery BenQ Medical Center The Affiliated BenQ Hospital of Nanjing Medical University Nanjing People's Republic of China; ^3^ Department of General Surgery Shanghai General Hospital Shanghai Jiao Tong University School of Medicine Shanghai People's Republic of China; ^4^ Singleron Biotechnologies Nanjing Jiangsu People's Republic of China; ^5^ Research Institute of General Surgery Jinling Hospital Jinling Clinical Medical College Nanjing University of Chinese Medicine Nanjing People's Republic of China

**Keywords:** CD38, *Klebsiella pneumoniae*, nicotinamide, scH16S‐seq, sepsis

## Abstract

Sepsis arises from heterogeneous host‐cell states that influence bacterial clearance, yet single‐cell transcriptomic approaches do not directly connect microbial signals with host transcriptional programs. This study introduces single‐cell host–bacterial 16S co‐sequencing (scH16S‐seq), which couples host transcriptomes with barcode‐resolved bacterial 16S‐derived UMI signals to resolve bacterial signal‐enriched host‐cell states. Applied to *Klebsiella pneumoniae* (KP) sepsis, scH16S‐seq mapped cell‐associated bacterial 16S‐derived signals to discrete *CD38^+^* myeloid states, including Acod1^+^ monocyte‐derived macrophages, *Lyve1* ^+^ interstitial macrophages, and hypoxic neutrophils sharing inflammatory, hypoxic, and metabolically stressed programs. Increased myeloid CD38 expression is also observed across human sepsis cohorts. Mechanistically, KP induces CD38‐associated NAD^+^depletion, mitochondrial bioenergetic failure, reduced ATP, and impaired lysosomal acidification. CD38 inhibition, NAD‐related metabolic intervention, or *Cd38* deficiency restores NAD homeostasis and mitochondrial function, whereas blockade of mitochondrial ATP synthesis or V‐ATPase‐dependent acidification attenuates lysosomal and antibacterial rescue. Lyz2‐Cre‐mediated *Cd38* deletion improves bacterial control and sepsis outcomes in vivo. Together, scH16S‐seq resolves KP 16S signal‐enriched myeloid states and identifies a targetable CD38–NAD–mitochondrial–lysosomal axis linking immunometabolic dysfunction to impaired antibacterial defense in sepsis.

## Graphical abstract

Single‐cell host–bacterial 16S co‐sequencing (scH16S‐seq) integrates cell‐associated bacterial 16S‐derived signals with host transcriptomes to resolve bacterial signal‐enriched myeloid states in *Klebsiella pneumoniae* sepsis. Complementary pharmacological and genetic analyses define a CD38–NAD immunometabolic axis connecting mitochondrial bioenergetic failure with impaired lysosomal acidification and defective bacterial clearance, revealing a targetable pathway for host‐directed intervention.

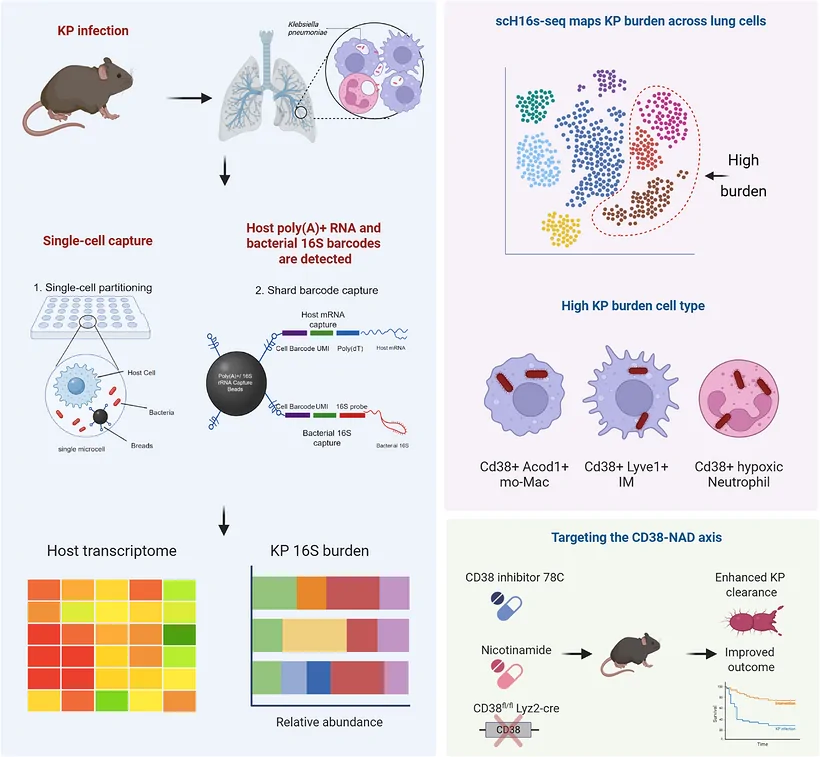

## 1 Introduction

Sepsis is a life‐threatening syndrome caused by a dysregulated host response to infection [1]. Sepsis severity is shaped not only by pathogen burden, but also by the long‐standing host–pathogen arms race in which bacteria may exploit or reshape host immune programs in ways that favor survival within infected tissues [2]. *Klebsiella pneumoniae* (KP) is a clinically important Gram‐negative opportunistic pathogen that causes severe bloodstream infection and sepsis, with limited therapeutic options [3, 4]. Although neutrophils and monocyte–macrophage populations are central to pulmonary antibacterial defense, KP can remodel innate immune responses to promote infection by subverting host cell metabolism [5, 6]. Thus, a key unresolved question is how to locate, among the many inflammatory cells present in infected tissue, the host‐cell states most closely associated with bacterial signals, and whether the genes and pathways defining these cells can reveal host‐directed strategies to improve bacterial clearance.

This gap is particularly relevant in KP sepsis, where lung myeloid cells are highly heterogeneous [7, 8]. Prior in vivo analyses have suggested that KP can associate with and reprogram lung interstitial macrophage populations [5]. Yet it remains unclear whether KP‐associated bacterial signals concentrate within specific macrophage or neutrophil states and which molecular programs define these bacterial signal‐enriched states.

Single‐cell RNA sequencing has transformed the analysis of immune heterogeneity during infection, but it provides limited direct information on bacterial 16S‐derived signals. In contrast, bulk 16S sequencing quantifies microbial composition or abundance at the sample level but loses host cell identity. Recent approaches, including scDual‐Seq, INVADEseq, and microbial read‐mining strategies, have begun to connect host and microbial information at single‐cell resolution [9, 10, 11, 12, 13]. However, infected tissues still require approaches that assign bacterial 16S‐derived signals to host cell identity, transcriptional state, and functional outcome within the same analytical framework.

CD38–NAD metabolism has been implicated in immune activation and antibacterial responses [14, 15, 16]. CD38 is a multifunctional immune ectoenzyme with NADase activity that can constrain NAD^+^ availability and reshape immune‐metabolic signaling [14, 17]. NAD^+^ metabolism is closely coupled to mitochondrial redox homeostasis and oxidative phosphorylation, whereas lysosomal acidification depends largely on ATP‐driven V‐ATPase proton pumping and is essential for phagolysosomal microbial degradation [18, 19, 20].

Here, we applied single‐cell host–bacterial 16S co‐sequencing, hereafter referred to as scH16S‐seq, to map KP 16S‐derived UMI signals onto host lung‐cell states during KP infection. This approach identified *Cd38* ^+^ *Acod1* ^+^ monocyte‐derived macrophages, *Cd38* ^+^ *Lyve1* ^+^ interstitial macrophages, and *Cd38* ^+^ hypoxic neutrophils as recurrent KP UMI‐enriched myeloid states. Human sepsis datasets further supported increased CD38 expression in circulating monocytes. Functionally, CD38‐targeted and NAD‐related interventions promoted intracellular KP clearance and improved infection outcomes in vivo. Together, our findings establish a cell‐resolved framework for linking bacterial 16S‐derived signals to host myeloid states.

## 2 Results

### 2.1 scH16S‐Seq Enables Barcode‐Resolved Host Transcriptome and Bacterial 16S UMI Co‐Profiling

To resolve host cell states associated with bacterial signals at single‐cell resolution during KP infection, we co‐developed and applied a FocuSCOPE 16S‐based single‐cell host–bacterial co‐sequencing strategy, hereafter referred to as scH16S‐seq. This approach couples host mRNA capture with bacterial 16S‐targeted detection through a shared single‐cell barcode, allowing the interpretation of bacterial 16S‐derived UMI signals alongside host cell gene expression (Figure 1A,B). Compared with microbial read mining from conventional scRNA‐seq and existing host–microbe co‐profiling methods, the scH16S‐seq workflow incorporates host transcriptome profiling, bacterial 16S‐targeted capture, staged 16S enrichment, and barcode‐resolved host–bacterial linkage within a unified workflow. Specifically, bacterial 16S‐derived signals are first retained within the barcode‐associated single‐cell capture and early amplification framework, followed by dedicated 16S library enrichment, thereby enabling cell‐resolved assessment of bacterial 16S‐derived UMI signals in parallel with host transcriptional states (Figure S1A).

**FIGURE 1:**
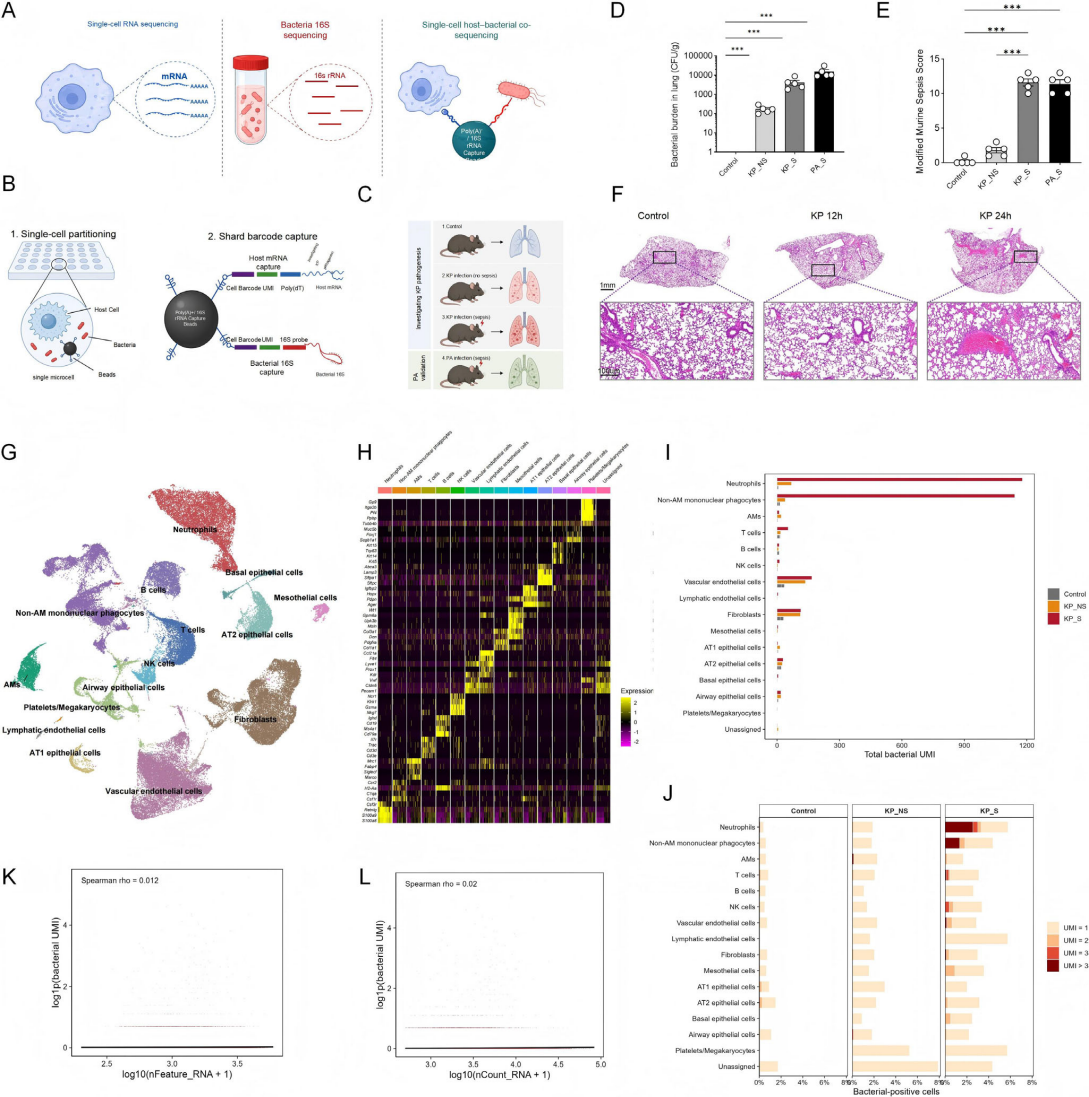
scH16S‐seq Maps Cell‐associated Bacterial 16S‐derived UMI Signals Onto Host Lung‐cell States During KP‐induced Sepsis. (A) Conceptual comparison of conventional single‐cell RNA sequencing, bulk 16S rRNA sequencing, and FocuSCOPE 16S‐based single‐cell host–bacterial 16S co‐sequencing, referred to here as scH16S‐seq. (B) Schematic of the FocuSCOPE 16S‐based scH16S‐seq strategy, showing single‐cell partitioning and shared barcode capture of host mRNA and bacterial 16S‐derived signals. (C) Experimental design. Control mice, *Klebsiella pneumoniae* non‐septic infection mice (KP_NS), and *Klebsiella pneumoniae* septic infection mice (KP_S) were used to define the KP infection axis. *Pseudomonas aeruginosa* septic infection mice (PA_S) were included as a supportive Gram‐negative infection comparison. (D–E) Pulmonary bacterial burden was measured by colony‐forming unit (CFU) assay (D) and clinical sepsis scores (E), used to characterize infection severity across the indicated experimental groups. (F) Representative H&E‐stained lung sections from Control, KP_NS (KP12h), and KP_S (KP24h) mice. (G) UMAP embedding of more than 70,000 integrated lung cells, colored by major cell‐type annotation. (H) Heatmap of canonical lineage markers supporting major cell‐type annotations. (I) Distribution of KP‐associated 16S‐derived UMI signals across major lung cell types in Control, KP_NS, and KP_S groups. (J) Load‐stratified frequencies of KP 16S UMI‐positive cells across major lung cell types and sample groups. KP 16S UMI‐positive cells were stratified as UMI = 1, UMI = 2, UMI = 3, and UMI > 3. (K, L) Technical‐control analysis showing correlations between log1p‐transformed KP 16S‐derived UMI counts and log10‐transformed host nFeature_RNA (K) or nCount_RNA (L). Spearman correlation coefficients are shown.

### 2.2 scH16S‐Seq Maps Cell‐Associated KP 16S‐Derived UMI Signals Across Lung Cell Types

We next applied scH16S‐seq to a systemic infection model established with KP, a clinically important Gram‐negative pathogen associated with sepsis [3, 4]. *Pseudomonas aeruginosa* (PA) septic infection was included as a supportive Gram‐negative infection comparison. Mice were classified as Control, KP non‐septic (KP_NS), KP septic (KP_S), or PA septic (PA_S) based on pulmonary bacterial burden and murine sepsis score (Figure 1C–E). Consistent with greater disease severity, lung injury scores were significantly higher in KP_S mice than in Control and KP_NS mice (Figure 1F, Figure S1B).

After quality control and integration, over 70 000 lung cells were retained for downstream analysis (Figure S1C). Unsupervised embedding resolved major immune, stromal, endothelial, and epithelial compartments, including neutrophils, non‐alveolar macrophage mononuclear phagocytes, alveolar macrophages, T cells, B cells, NK cells, endothelial cells, fibroblasts, epithelial cells, and platelets/megakaryocytes (Figure 1G, Figure S1D,E). Canonical marker expression supported these annotations, with alveolar macrophages marked by Siglecf, Marco and Fabp4, and non‐alveolar mononuclear phagocytes marked by *Csf1r*, *Lyz2* and *Ccr2* (Figure 1H).

We next mapped KP 16S‐derived UMI signals across the annotated host‐cell landscape (Figure 1I). In the control dataset, KP 16S‐derived signals were detected at a low baseline level. In the pooled KP_S dataset, KP‐associated signals were more abundant and were preferentially concentrated in neutrophils and non‐alveolar mononuclear phagocytes, which together accounted for the dominant fraction of the annotated KP bacterial UMI burden (Figure 1I,J). This distribution is consistent with the established roles of neutrophils and recruited monocyte–macrophage populations in pulmonary antibacterial defense against Gram‐negative pathogens [5, 6].

We further assessed bacterial‐signal recovery in computationally defined background barcodes. In KP_S, KP‐reference‐aligned UMI density was enriched 52.5‐fold in called‐cell relative to background barcodes. Similarly, in the supportive PA_S dataset, PA‐reference‐aligned UMI density showed a 22.2‐fold enrichment in called‐cell barcodes (Table S1). Target‐reference mapping further showed that 92.48% of KP reads and 98.86% of PA reads were uniquely mapped among target‐mapped reads before feature assignment (Table S2). Together with the low baseline bacterial signal in the control dataset, these analyses show that bacterial 16S‐derived signals were preferentially concentrated in called‐cell barcodes, with relatively limited mapping ambiguity among reads aligned to the predefined bacterial target references.

Because bacterial UMI capture could be influenced by library complexity, we next examined the relationship between KP UMI counts and host RNA quality metrics. Across lung cells in the KP analysis, Spearman correlations between log1p (KP bacterial UMI) and either log10 (nCount_RNA ^+^ 1) or log10 (nFeature_RNA ^+^ 1) were close to zero (rho = 0.020 and 0.012, respectively; Figure 1K,L). These results suggest that the bacterial UMI signal was not primarily driven by host RNA library size or detected gene complexity.

The supportive PA_S dataset showed a similar pattern. PA‐associated bacterial UMI signals were also mainly detected in neutrophils and non‐alveolar mononuclear phagocytes (Figure S1F–J), with similarly weak correlations between PA UMI counts and host nCount_RNA or nFeature_RNA (rho = 0.064 and 0.032, respectively; Figure S1H). Together, these data support scH16S‐seq as a cell‐resolved framework for linking bacterial 16S‐derived UMI signals to host‐cell identity and gene expression during infection.

### 2.3 Cd38^+^ Acod1^+^ Mo‐Macs and Cd38^+^ Lyve1^+^ IMs are Enriched for KP 16S‐Derived UMI Signals

We next subclustered lung mononuclear phagocytes, a compartment composed of anatomically and functionally distinct alveolar macrophages, interstitial macrophages, recruited monocyte‐derived macrophages, and DC subsets [5, 7, 21]. This analysis identified *Cd38* ^+^ *Acod1* ^+^ mo‐Macs, *S100a8/9* ^+^ monocytes, *Cd38* ^+^ *Lyve1* ^+^ IMs, *MHC‐II* ^+^ IMs, AMs and DC subsets (Figure 2A–C). Canonical markers confirmed these annotations, distinguishing monocyte/mo‐Mac states, IMs, AMs, and DC lineages (Figure 2D).

**FIGURE 2:**
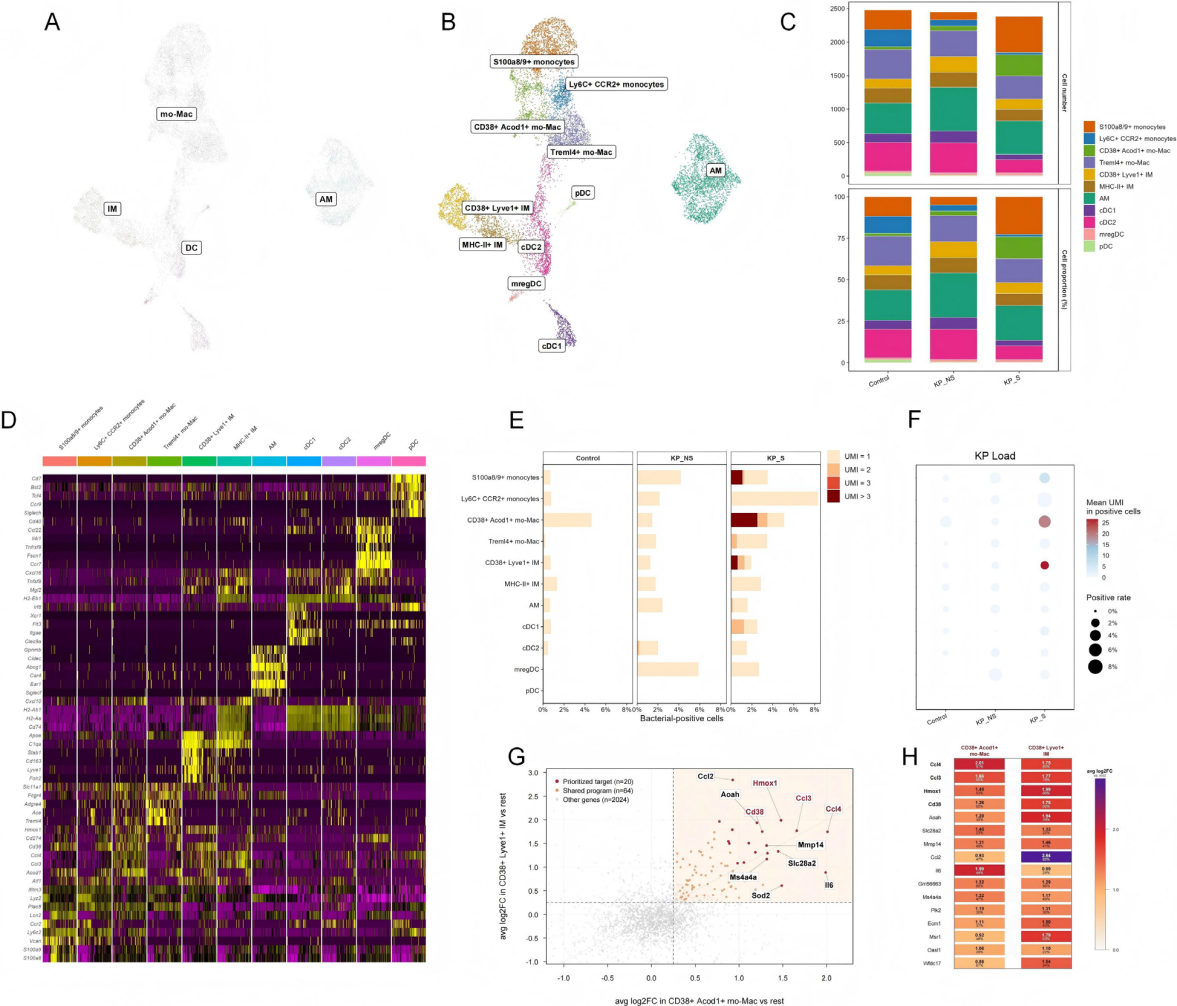
*Cd38* ^+^ *Acod1* ^+^ mo‐Macs and *Cd38* ^+^ *Lyve1* ^+^ IMs Are Enriched for KP 16S‐derived UMI Signals During KP Sepsis. (A) UMAP of the reclustered mononuclear phagocyte compartment, annotated into broad states including monocyte‐derived macrophages (mo‐Macs), interstitial macrophages (IMs), alveolar macrophages (AMs), and dendritic cells (DCs). (B) UMAP of fine‐state annotations within the reclustered mononuclear phagocyte compartment. (C) Cell numbers and proportional composition of fine mononuclear phagocyte states across Control, KP_NS, and KP_S groups. (D) Heatmap of canonical markers supporting fine‐state annotation. (E) Load‐stratified frequencies of KP 16S UMI‐positive cells across fine mononuclear phagocyte states. (F) Bubble plot showing KP 16S‐derived UMI enrichment across fine mononuclear phagocyte states. Bubble size indicates the KP 16S UMI‐positive rate, and color indicates the mean KP 16S‐derived UMI count among positive cells. (G) State‐enriched marker‐gene profiles comparing *Cd38* ^+^ *Acod1* ^+^ mo‐Macs with other monocyte/mo‐Mac states and *Cd38* ^+^ *Lyve1* ^+^ IMs with other IM states. (H) Heatmap of prioritized genes enriched in *Cd38* ^+^ *Acod1* ^+^ mo‐Macs and *Cd38* ^+^ *Lyve1* ^+^ IMs relative to their respective comparator states.

Mapping KP bacterial UMI signals onto these subpopulations revealed that KP UMI‐positive cells were unevenly distributed across the compartment in KP_S lungs (Figure 2E,F). *Cd38* ^+^ *Acod1* ^+^ mo‐Macs and *Cd38* ^+^ *Lyve1* ^+^ IMs showed relatively high KP UMI‐positive fractions and the highest mean KP bacterial UMI burden (Figure 2E,F). These data identify *Cd38* ^+^ *Acod1* ^+^ mo‐Macs and *Cd38* ^+^ *Lyve1* ^+^ IMs as the macrophage states most strongly associated with KP 16S‐derived UMI signals during KP infection. We then compared the transcriptional programs of these two states. *Cd38* ^+^ *Acod1* ^+^ mo‐Macs and *Cd38* ^+^ *Lyve1* ^+^ IMs shared an inflammatory and stress‐response signature, including *Ccl3*, *Ccl4*, *Hmox1*, *Cd38*, *Aoah*, *Mmp14* and *Slc28a2* (Figure 2G,H).

In the supportive PA cohort, PA UMI signals were broadly distributed across mononuclear phagocyte states, but *Cd38* ^+^ *Lyve1* ^+^ IMs and *Cd38* ^+^ *Acod1* ^+^ mo‐Macs again showed relatively high PA UMI‐positive fractions and bacterial UMI burden (Figure S2A–C).

These states did not show disproportionate increases in detected gene number, total host RNA UMI counts, or mitochondrial transcript percentage, suggesting that bacterial UMI enrichment was not primarily explained by library complexity or cell quality (Figure S2D).

### 2.4 Cd38^+^ Hypoxic Neutrophils are Enriched for KP 16S‐Derived UMI Signals

We next examined neutrophil heterogeneity during KP infection, which was less complex than the macrophage landscape. Reclustering identified six neutrophil states: *Cd38* ^+^ hypoxic, inflammatory, ISG‐high, *Ltf* ^+^ degranulating, *Alox5* ^+^ lipid‐remodeling, and *Olfm4* ^+^ immature neutrophils (Figure 3A,B). Neutrophil‐state composition differed across the pooled KP datasets (Figure 3C), and canonical marker expression supported these annotations (Figure 3D).

**FIGURE 3:**
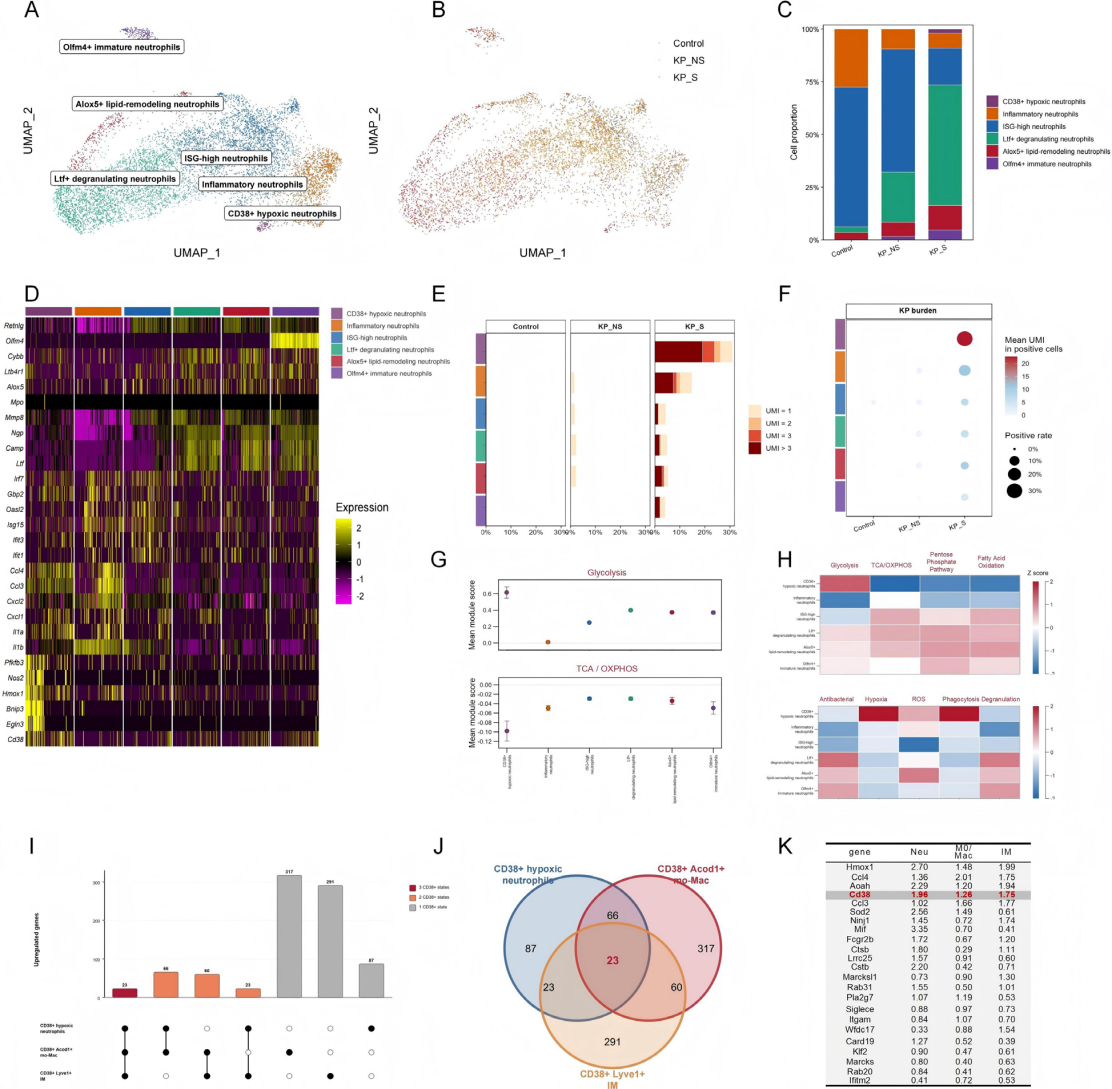
CD38 Marks Convergent Bacterial 16S UMI‐enriched Myeloid States During KP Sepsis. (A) UMAP of reclustered neutrophils resolved into six transcriptionally distinct states. (B) UMAP of reclustered neutrophils colored by KP discovery‐axis sample group. (C) Neutrophil state composition across Control, KP_NS, and KP_S groups. (D) Heatmap of marker genes supporting neutrophil state annotation. (E) Load‐stratified frequencies of KP 16S UMI‐positive cells across neutrophil states. (F) Bubble plot showing KP 16S‐derived UMI enrichment across neutrophil states. Bubble size indicates the KP 16S UMI‐positive rate, and color indicates the mean KP 16S‐derived UMI count among positive cells. (G) Glycolysis and TCA/OXPHOS module scores across neutrophil states. (H) Heatmaps of metabolic and functional module scores across neutrophil states. (I) UpSet plot showing the overlap of upregulated marker genes among *Cd38* ^+^ hypoxic neutrophils, *Cd38* ^+^ *Acod1* ^+^ mo‐Macs, and *Cd38* ^+^ *Lyve1* ^+^ IMs. (J) Venn diagram summarizing shared and state‐restricted upregulated marker genes across the three *Cd38* ^+^ myeloid states. (K) Prioritized shared marker genes across the three *Cd38* ^+^ myeloid states, with average log2 fold change shown for each state.

Mapping KP bacterial UMI signals onto neutrophil states revealed a state‐selective distribution. *Cd38* ^+^ hypoxic neutrophils showed the greatest enrichment of high‐load KP UMI‐positive cells and the greatest mean KP bacterial UMI burden (Figure 3E,F). Gene‐module scoring showed higher glycolysis scores and lower TCA/OXPHOS scores in *Cd38* ^+^ hypoxic neutrophils (Figure 3G,H), consistent with a hypoxia‐associated metabolic transcriptional program. Notably, despite carrying the highest bacterial UMI burden, *Cd38* ^+^ hypoxic neutrophils showed relatively lower antibacterial and degranulation module scores than *Ltf^+^* degranulating, *Alox5* ^+^ lipid‐remodeling, and *Olfm4* ^+^ immature neutrophils (Figure 3H). This uncoupling between KP 16S‐derived UMI enrichment and canonical antimicrobial effector modules suggests that *Cd38* ^+^ hypoxic neutrophils represent a bacterial signal‐enriched neutrophil state with reduced antimicrobial transcriptional features, raising the possibility of impaired bacterial processing.

We hypothesized that shared marker genes in these high bacterial UMI‐burden cell states may be linked to putative bacterial‐processing defects and bacterial 16S‐derived signal accumulation. We then compared *Cd38* ^+^ hypoxic neutrophils, *Cd38* ^+^ *Acod1* ^+^ mo‐Macs and *Cd38* ^+^ *Lyve1* ^+^ IMs. Upset and Venn analyses identified 23 genes shared across all three states (Figure 3I,J), including *Cd38*, *Hmox1*, *Ccl3*, *Ccl4*, *Aoah*, *Sod2*, *Ninj1* and *Mif* (Figure 3K). Gene‐enrichment analysis across all three states revealed a shared transcriptional program, including immune signal transduction, antibacterial responses, hypoxia, and oxidative‐stress‐associated pathways (Figure S3A).

In the supportive PA cohort, PA bacterial UMI signals again accumulated within neutrophil states, with *Cd38* ^+^ hypoxic neutrophils showing the strongest PA UMI burden and high‐load PA UMI enrichment (Figure S3B–D). QC distributions of detected genes, total host RNA UMI counts, and mitochondrial transcript percentage did not mirror the bacterial UMI hierarchy (Figure S3E).

To provide orthogonal spatial validation, KP 16S‐FISH was combined with CD38 and CD11b staining in lung sections. The majority of KP‐FISH^+^CD11b^+^ cells were CD38^+^ in infected lungs, providing independent spatial support for the CD38‐high bacterial signal‐enriched myeloid phenotype identified by scH16S‐seq (Figure S3F,G).

Together, using scH16S‐seq, we identified CD38 as a recurrent marker of bacterial UMI‐enriched inflammatory myeloid states in KP‐induced sepsis.

### 2.5 Cross‐Modal and Cross‐Cohort Human Datasets Support Increased Myeloid CD38 Expression in Sepsis

To assess whether CD38 upregulation is conserved in human sepsis, we first analyzed the adult CITE‐seq cohort GSE279451, which profiled paired transcriptomes and surface proteins in PBMCs from healthy controls and patients with sepsis [22, 23]. Focusing on the sepsis vs. healthy‐control comparison, we generated donor‐level pseudobulk RNA and antibody‐derived tag (ADT) profiles (Figure 4A). Among 42 matched RNA–ADT features, CD38 showed concordant increases at both the RNA and protein levels (Figure 4B). We next examined CD38 expression in purified peripheral‐blood CD14^+^ monocytes from three independent GEO cohorts (Figure 4C) [24]. *Cd38* expression was consistently higher in sepsis across all three CD14^+^ monocyte datasets (Figure 4D), supporting increased monocyte *Cd38* expression as a reproducible feature of human sepsis.

**FIGURE 4:**
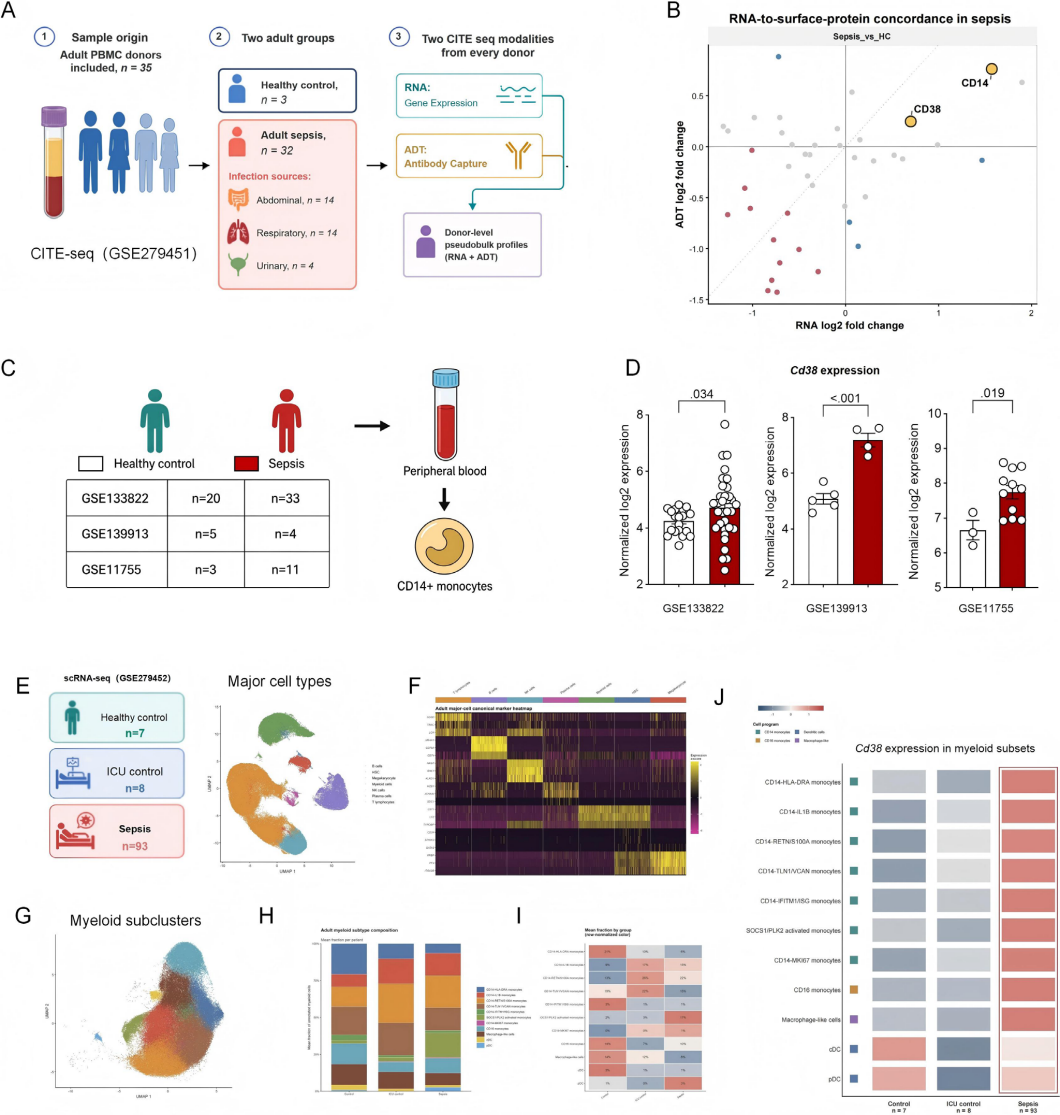
Cross‐modal and Cross‐cohort human Datasets Support Increased Myeloid CD38 Expression in sepsis. (A) Overview of the adult CITE‐seq cohort GSE279451. Donor‐level pseudobulk RNA and antibody‐derived tag (ADT) profiles were generated for the sepsis vs. healthy‐control comparison. (B) Concordance between sepsis‐associated RNA and surface‐protein changes for 42 matched RNA–ADT features in GSE279451. CD14 and CD38 are highlighted. (C) Validation strategy using purified peripheral‐blood CD14^+^ monocytes from three independent GEO cohorts: GSE133822, GSE139913, and GSE11755. Only healthy‐control and sepsis samples were included. (D) Normalized log2 CD38 expression in CD14^+^ monocytes from the three validation cohorts. (E) Overview of the GSE279452 cohort. Left, cohort composition. Right, Harmony‐integrated UMAP of adult PBMCs from GSE279452, colored by major cell type. The analysis included healthy controls (*n* = 7), ICU controls without sepsis (*n* = 8), and patients with sepsis (*n* = 93). (F) Canonical marker heatmap supporting major immune‐cell annotations. (G) Harmony‐integrated UMAP of reclustered myeloid cells, colored by 11 annotated myeloid states. (H) Mean per‐donor composition of myeloid states across healthy controls, ICU controls, and patients with sepsis. (I) Heatmap summary of the group means shown in H. Values indicate the mean percentage of each state among resolved myeloid cells. Color is scaled independently within each state. (J) Mean sample‐level CD38 expression across adult myeloid states. CD38 counts were library‐size normalized and log‐transformed at the cell level, averaged within each donor and state, and then averaged by clinical group. Color is scaled independently within each state; comparisons should therefore be interpreted horizontally within rows.

Finally, we analyzed adult PBMC scRNA‐seq profiles from GSE279452, including healthy controls, ICU controls, and patients with sepsis (Figure 4E). Major immune compartments were resolved, including T cells, B cells, NK cells, plasma cells, and myeloid cells (Figure 4F). Lineage‐specific reclustering of myeloid cells identified 11 transcriptionally distinct states (Figure 4G–I). Consistent with a recent single‐cell multi‐omic sepsis atlas, *Cd38* expression showed a coordinated increase across most myeloid cells in patients with sepsis (Figure 4J). Together, these cross‐modal, bulk‐sorted and single‐cell analyses support CD38 as a clinically relevant marker of sepsis‐associated monocyte activation in humans.

### 2.6 CD38‐Targeted and NAD‐Related Interventions Dampen KP‐Induced Myeloid Activation and Inflammatory Cytokine Responses

We next examined whether KP induces CD38 expression in vivo and in macrophages. Immunofluorescence analysis of KP‐infected iBMDMs showed a marked increase in CD38 signal compared with uninfected controls (Figure S4A,B). To provide protein‐level validation, we performed immunoblotting in lung tissue collected 24 h after systemic KP infection. CD38 protein abundance was significantly increased in KP‐infected lungs compared with controls (Figure S4C,D). Consistently, immunoblotting of BMDMs showed a progressive increase in CD38 protein during the first 3 h of KP infection (Figure S4E). Flow cytometry further confirmed increased CD38 expression in KP‐infected macrophages (Figure S4F). Together, these complementary assays demonstrate KP‐associated induction of CD38 at the protein level both in vivo and in macrophages.

Because CD38 is a major NAD^+^‐consuming ectoenzyme that links NAD^+^ availability to immunometabolic signaling [25, 26], we next tested two mechanistically distinct interventions targeting this axis: 78C, a pharmacological CD38 inhibitor, and nicotinamide (NAM), an NAD‐related metabolic intervention. Because NAM can affect multiple NAD‐dependent pathways, it was not interpreted as a CD38‐specific inhibitor. Both 78C and NAM attenuated the KP‐induced increase in CD38 signal detected by immunofluorescence and flow cytometry (Figure S4A,B,F), suggesting that CD38 expression is coupled to the inflammatory and metabolic activation state of infected macrophages.

We then assessed the broader inflammatory response. Transcriptomic profiling showed that KP induced chemokine‐, cytokine‐, and interferon‐related inflammatory programs, which were broadly attenuated by 78C and NAM treatment (Figure S4G). Consistently, KP‐induced *Il1b*, *Ifnb1*, and *Il6* expression in primary BMDMs was reduced by these interventions (Figure S4H). In PMA‐differentiated human THP‐1 cells, KP increased CD38 expression (Figure S4I) and robustly induced *Il6* and *Ifnb1*, whereas 78C and NAM attenuated the inflammatory cytokine responses (Figure S4J). Together, these findings show that KP induces CD38 across complementary experimental systems and that pharmacological modulation of the CD38–NAD axis dampens KP‐induced myeloid inflammatory activation.

### 2.7 CD38–NAD Modulation Restores NAD Homeostasis and Mitochondrial Bioenergetics During KP Infection

Because CD38 is a major NAD^+^‐consuming enzyme and NAD^+^ availability is closely linked to mitochondrial redox metabolism [27], we next directly examined whether KP‐induced CD38 upregulation was associated with disruption of cellular NAD homeostasis and mitochondrial bioenergetics [18, 19, 20, 28]. KP infection markedly reduced intracellular NAD^+^ abundance and the NAD^+^/NADH ratio, accompanied by a pronounced decrease in cellular ATP. CD38 inhibition with 78C, NAD‐related metabolic intervention with NAM, and *Cd38* deficiency restored these metabolic parameters to varying degrees (Figure 5A–C).

**FIGURE 5:**
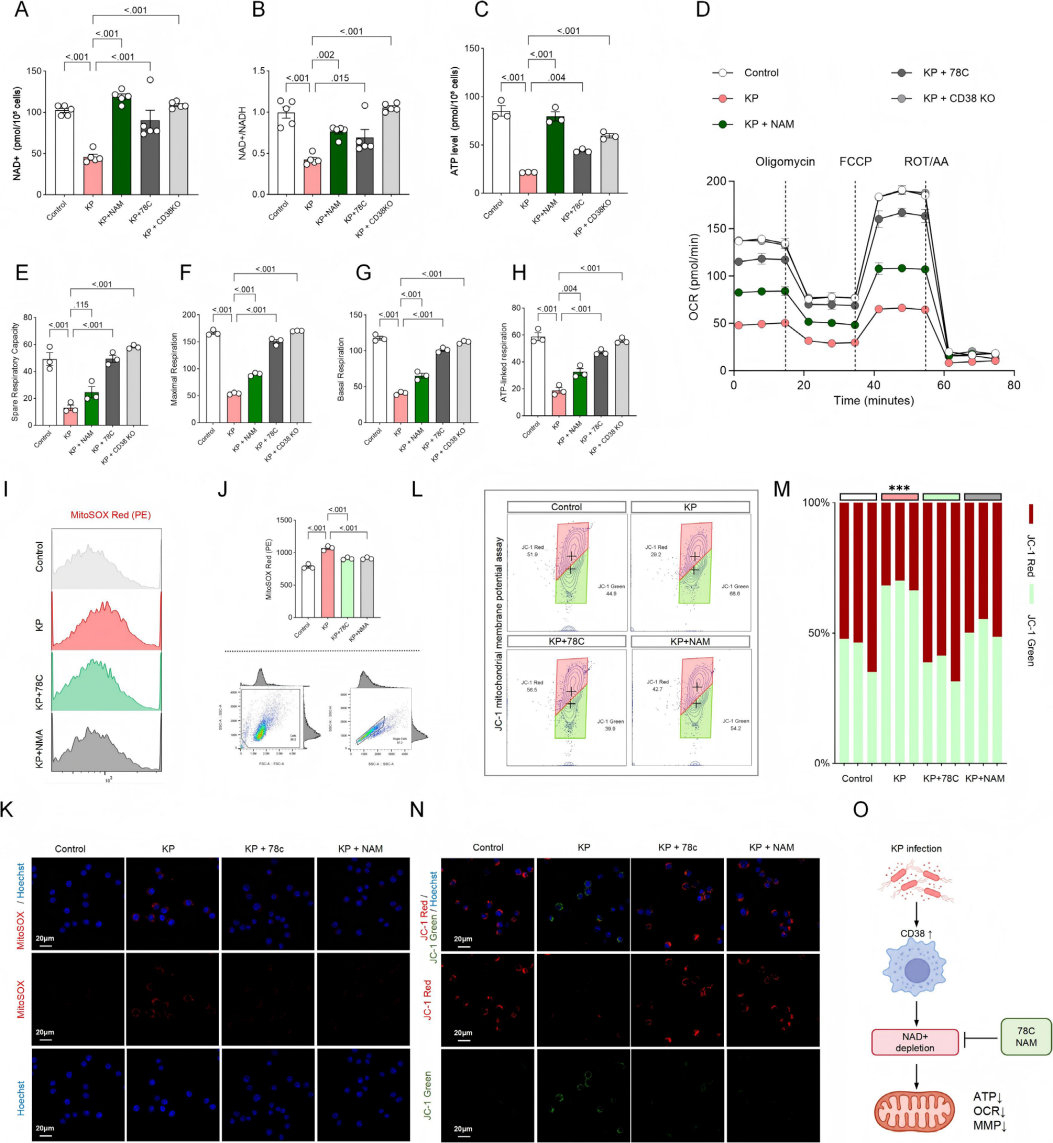
CD38–NAD Modulation Restores NAD Homeostasis and Mitochondrial Bioenergetics During KP Infection. (A) Intracellular NAD^+^ abundance in Control and KP‐infected macrophages with or without NAM treatment, CD38 inhibition with 78C, or *CD38* deficiency. NAD^+^ abundance was normalized to cell number and expressed as pmol per 10^5^ cells. (B) Intracellular NAD^+^/NADH ratio in the indicated groups. (C) Intracellular ATP abundance in the indicated groups, normalized to cell number and expressed as pmol per 10^5^ cells. (D) Representative oxygen‐consumption rate (OCR) profiles obtained by Seahorse mitochondrial stress testing. Oligomycin, FCCP, and rotenone/antimycin A (ROT/AA) were sequentially injected at the indicated time points. (E–H) Quantification of spare respiratory capacity (E), maximal respiration (F), basal respiration (G), and ATP‐linked respiration (H) derived from the Seahorse mitochondrial stress test. (I) Representative flow cytometry histograms of MitoSOX Red fluorescence in iBMDMs infected with live KP for 3 h at MOI 50, with or without 78C pretreatment (2 µM, 1 h) or NAM pretreatment (2 mM, 4 h). (J) Quantification of MitoSOX Red fluorescence intensity in the groups shown in I; representative flow cytometry gating is shown below. (K) Representative fluorescence images of MitoSOX Red and Hoechst in iBMDMs across the indicated treatment groups. Scale bars, 20 µm. (L) Representative JC‐1 flow cytometry plots assessing mitochondrial membrane potential in Control and KP‐infected iBMDMs with or without 78C or NAM pretreatment. JC‐1 red aggregates indicate polarized mitochondria, whereas increased JC‐1 green monomers indicate mitochondrial depolarization. (M) Quantification of JC‐1 red and green fractions across the indicated groups. (N) Representative JC‐1 fluorescence images showing JC‐1 red, JC‐1 green, and Hoechst signals across treatment groups. Scale bars, 20 µm. (O) Proposed model summarizing the CD38–NAD–mitochondrial bioenergetic axis.

Transcriptomic analysis showed that KP infection suppressed a mitochondrial respiratory‐chain program, with prominent reduction of complex IV‐associated genes, including *COX1*, *COX2*, and *COX3* (Figure S5A). qPCR validation confirmed that KP reduced *COX1*, *COX2* and *COX3* expression, whereas 78C and NAM partially restored their expression (Figure S5B). We next directly assessed mitochondrial respiratory function using Seahorse mitochondrial stress testing. KP infection markedly suppressed oxygen‐consumption rate and reduced spare respiratory capacity, maximal respiration, basal respiration, and ATP‐linked respiration. CD38 inhibition partially restored these respiratory parameters, whereas NAM treatment and *Cd38* deficiency produced broader recovery of mitochondrial respiratory capacity (Figure 5D–H). Consistently, KP markedly increased mitochondrial superoxide signals by both flow cytometry and fluorescence imaging, while 78C and NAM reduced MitoSOX intensity (Figure 5I–K). JC‐1 analysis further showed that KP increased the depolarized green fraction and reduced the red/green balance, indicating loss of mitochondrial membrane potential; both 78C and NAM partially restored the JC‐1 red signal and reduced the depolarized green fraction (Figure 5L–N).

Together, these metabolic, respiratory, and mitochondrial phenotyping data support a model in which CD38‐associated NAD dysregulation contributes to mitochondrial bioenergetic failure during KP infection (Figure 5O).

### 2.8 Restored Mitochondrial Bioenergetics Supports V‐ATPase‐Dependent Lysosomal Acidification and Intracellular KP Clearance

We next asked whether restoration of mitochondrial bioenergetics was functionally linked to lysosomal acidification [18, 19, 20]. Because lysosomal acidification relies heavily on ATP‐driven V‐ATPase proton pumping, we hypothesized that rescuing NAD^+^‐mediated mitochondrial oxidative phosphorylation would restore the cellular energy required for this organelle function. As a positive control, CCCP, a protonophore that dissipates mitochondrial membrane potential and secondarily perturbs cellular energy‐dependent organelle homeostasis, markedly reduced both MitoTracker Red CMXRos and LysoTracker Green DND‐26 fluorescence (Figure S5C,D). KP infection similarly decreased CMXRos fluorescence and reduced LysoTracker Green DND‐26 intensity, indicating concomitant mitochondrial depolarization and lysosomal deacidification; both 78C and NAM restored these signals (Figure 6A–C). Ratiometric analysis using LysoSensor Yellow/Blue DND‐160 further confirmed that KP reduced the 540/440 nm ratio, consistent with impaired lysosomal acidification, whereas 78C and NAM partially restored this ratio (Figure 6D).

**FIGURE 6:**
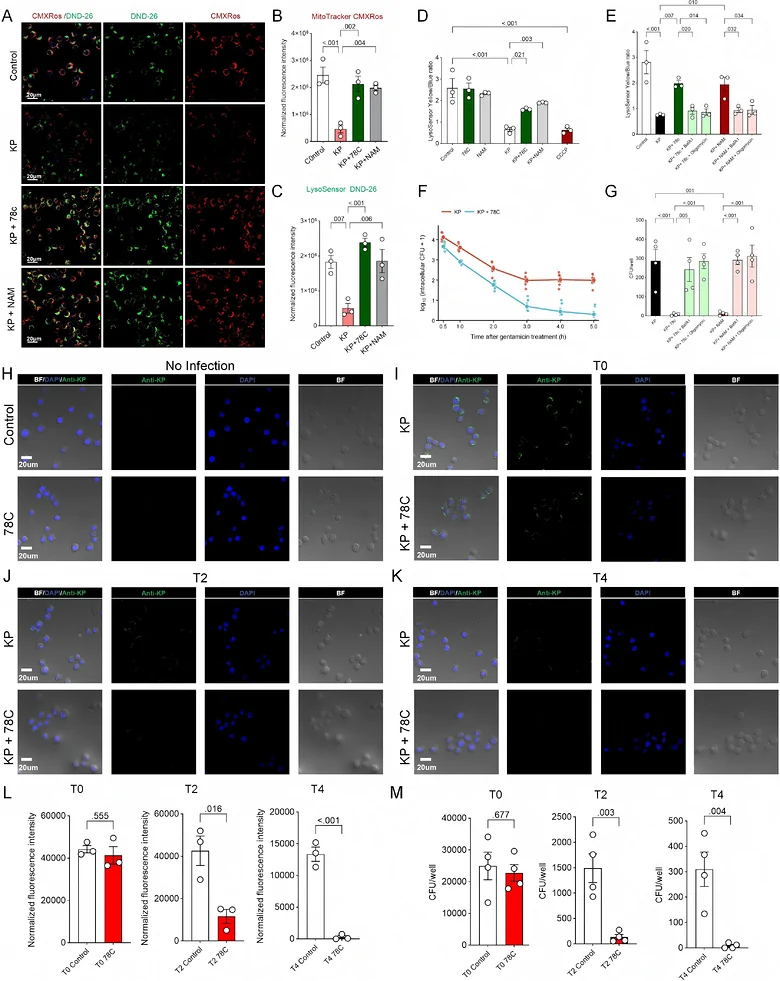
Restored Mitochondrial Bioenergetics Supports V‐ATPase‐dependent Lysosomal Acidification and Intracellular KP Clearance. (A) Representative live‐cell fluorescence images of MitoTracker Red CMXRos, LysoTracker Green DND‐26, and Hoechst in iBMDMs infected with live KP for 3 h at MOI 50, with or without 78C pretreatment (2 µM, 1 h) or NAM pretreatment (2 mM, 4 h). Cells were stained with CMXRos (50 nM) and LysoTracker Green DND‐26 (50 nM) for 15 min before imaging. Scale bars, 20 µm. (B, C) Quantification of normalized CMXRos (B) and LysoTracker Green DND‐26 (C) fluorescence intensity in the groups shown in A. (D) Ratiometric assessment of lysosomal acidification using LysoSensor Yellow/Blue DND‐160 in Control, 78C‐ or NAM‐treated uninfected cells and KP‐infected cells with or without 78C or NAM. CCCP was included as a lysosomal deacidification control. A higher Yellow/Blue fluorescence ratio indicates stronger lysosomal acidification. (E) LysoSensor Yellow/Blue analysis testing the dependence of the 78C‐ and NAM‐mediated lysosomal rescue on mitochondrial ATP production and V‐ATPase function. KP‐infected cells treated with 78C or NAM were additionally exposed to the mitochondrial ATP synthase inhibitor oligomycin or the V‐ATPase inhibitor bafilomycin A1 (BafA1). (F) Gentamicin‐protection assay showing viable intracellular KP over time in vehicle‐ and 78C‐treated macrophages. Following KP infection, extracellular bacteria were eliminated with gentamicin (50 µg/mL, 30 min), followed by culture in medium containing gentamicin (10 µg/mL). At the indicated time points, cells were lysed, and viable intracellular KP was quantified by CFU plating. Data are shown as log_10_(CFU + 1). (G) Gentamicin‐protection assay testing the dependence of the antibacterial effects of 78C and NAM on mitochondrial ATP production and V‐ATPase‐dependent lysosomal function. Viable intracellular KP was quantified in the indicated groups in the presence or absence of oligomycin or BafA1. (H) Representative anti‐KP immunofluorescence images of uninfected macrophages with or without 78C treatment, included as staining controls. KP is shown in green, DAPI in blue, and bright‐field (BF) images are shown on the right. (I–K) Representative anti‐KP immunofluorescence images of KP‐infected macrophages immediately after gentamicin protection (T0; I) and at 2 h (T2; J) and 4 h (T4; K) thereafter, with or without 78C treatment. (L) Quantification of normalized KP‐associated fluorescence intensity at T0, T2, and T4. (M) Parallel gentamicin‐protection CFU measurements at the corresponding time points. The initial post‐entry viable KP burden was comparable between groups at T0, whereas viable intracellular KP declined more rapidly following 78C treatment at T2 and T4.

We next tested whether the lysosomal rescue induced by CD38/NAD modulation required mitochondrial ATP production and intact V‐ATPase function. Inhibition of mitochondrial ATP synthase with oligomycin markedly attenuated the restoration of lysosomal acidification induced by either 78C or NAM. Similarly, inhibition of V‐ATPase with bafilomycin A1 substantially attenuated the lysosomal acidification rescue (Figure 6E). We then determined whether this mechanistic dependency translated into antibacterial function. In gentamicin‐protection assays, 78C accelerated the decline of viable intracellular KP over time (Figure 6F). Importantly, the reduction in intracellular viable KP induced by either 78C or NAM was markedly attenuated by oligomycin or bafilomycin A1 (Figure 6G), linking mitochondrial ATP production and V‐ATPase‐dependent lysosomal function to the enhanced bacterial clearance produced by CD38/NAD modulation.

To further resolve the temporal characteristics of this antibacterial effect, we performed time‐resolved anti‐KP fluorescence imaging together with parallel viable intracellular CFU measurements. Immediately after gentamicin protection (T0), intracellular KP‐associated fluorescence and viable CFU were comparable between control and 78C‐treated macrophages. At 2 and 4 h thereafter, however, KP‐associated fluorescence declined substantially more rapidly following CD38 inhibition (Figure 6H–L). Parallel CFU measurements showed the same temporal pattern, with significantly reduced viable intracellular KP in the 78C‐treated group at T2 and T4 (Figure 6M). These findings are consistent with accelerated post‐entry intracellular KP clearance following CD38 inhibition rather than simply reflecting differences in the initial post‐entry bacterial burden.

Together, these results support a mechanistic model in which CD38‐associated NAD depletion impairs mitochondrial bioenergetic capacity during KP infection, thereby limiting ATP‐dependent, V‐ATPase‐mediated lysosomal acidification and intracellular bacterial clearance. Restoration of CD38–NAD metabolism improves mitochondrial and lysosomal function, whereas blockade of mitochondrial ATP production or V‐ATPase activity disrupts this antibacterial rescue (Figure S5E).

### 2.9 CD38‐Targeted Intervention and Myeloid CD38 Deletion Attenuate KP‐Induced Sepsis and Bacterial Dissemination

KP infection increased pulmonary CD38 signal, and fixed‐tissue imaging showed that KP‐derived signals were frequently observed in spatial association with CD38‐rich regions in lung tissue (Figure 7A). Pharmacological modulation of the CD38/NAD‐related signaling axis with 78C or NAM improved survival and reduced clinical sepsis scores in KP‐infected mice (Figure 7B,C). Consistently, both interventions attenuated KP‐induced multi‐organ pathology, including intestinal injury, lung damage, and splenic architectural disruption (Figure 7D–G). Moreover, 78C and NAM reduced KP burden in the liver and lung, with a downward trend in the spleen (Figure 7H–J). In lung tissue, KP infection induced *Cd38* and Il6 expression; both interventions reduced *Cd38* expression, and 78C significantly suppressed Il6, with NAM showing a similar trend (Figure 7K,L).

**FIGURE 7:**
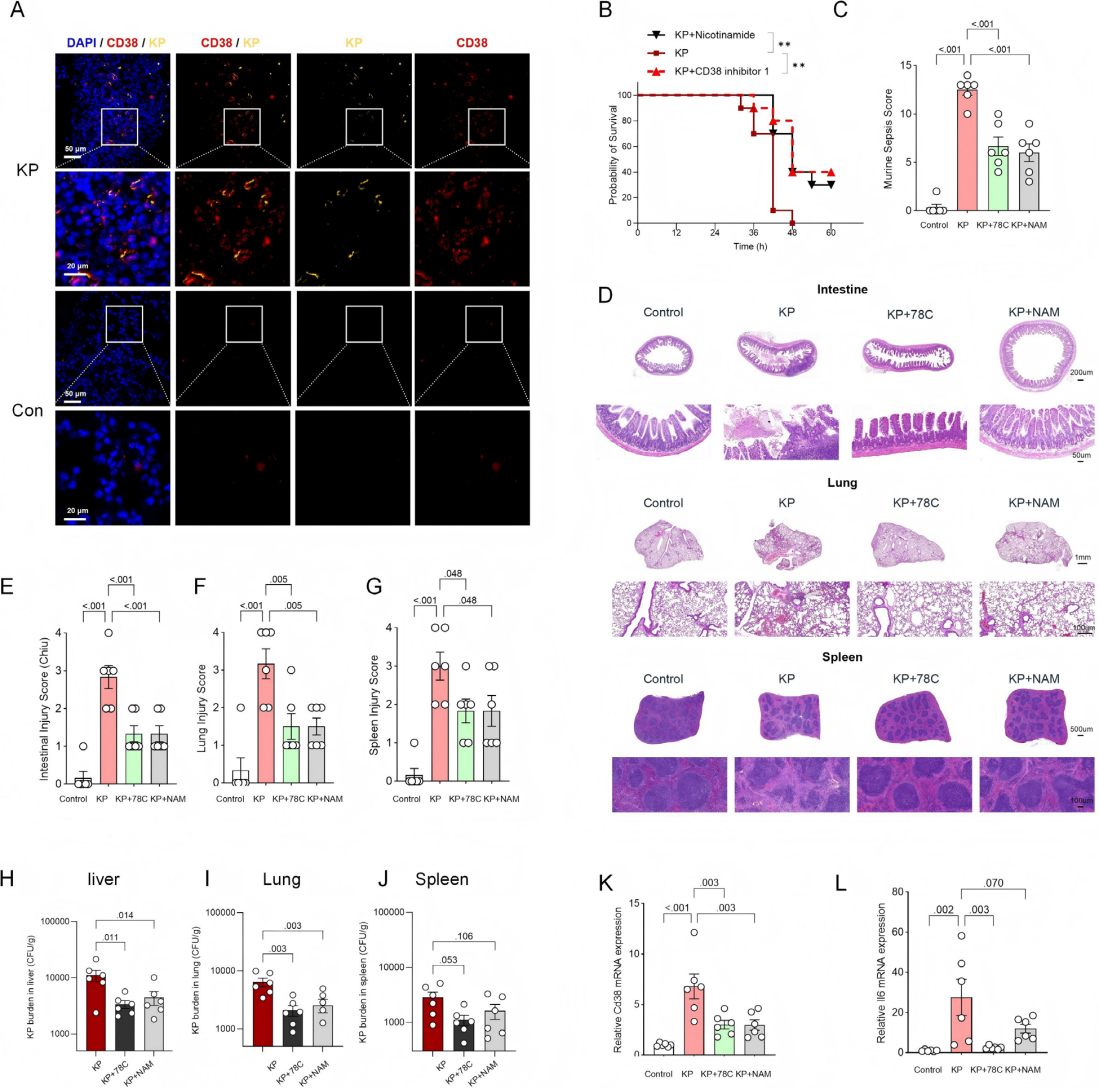
Pharmacological Modulation of CD38/NAD‐related Signaling Attenuates KP‐induced Sepsis and Bacterial Dissemination. (A) Representative immunofluorescence images showing CD38, KP, and DAPI signals in lung tissue from Control and KP‐infected mice. CD38 is shown in red, KP in yellow, and nuclei in blue. (B) Survival curves of KP‐infected mice treated with vehicle, 78C, or NAM. Mice were infected with KP at 1 × 10^3^ CFU, treated with 78C (15 mg/kg) or NAM (200 mg/kg) 1 h after infection, and monitored for 60 h. *n* = 10 mice per group. (C) Clinical sepsis scores of KP‐infected mice treated with vehicle, 78C, or NAM. Mice were infected with KP at 2 × 10^3^ CFU, treated as in B, and scored 24 h after infection. *n* = 6 mice per group. (D) Representative H&E‐stained sections of intestine, lung and spleen from Control, KP, KP+78C and KP+NAM groups. Tissues were collected 24 h after KP infection with 2 × 10^3^ CFU; 78C or NAM was administered 1 h after infection. *n* = 6 mice per group. (E–G) Quantification of intestinal injury, lung injury, and spleen injury scores at 24 h after KP infection. (H–J) KP bacterial burden in liver, lung, and spleen at 24 h after infection, quantified as colony‐forming units (CFU) per gram of tissue. (K, L) Relative *Cd38* and Il6 mRNA expression in lung tissue collected 24 h after KP infection. qPCR data were normalized to Actb.

To assess the contribution of CD38 across *Lyz2*‐expressing myeloid lineages in vivo, we next infected *Cd38* ^fl/fl^ *Lyz2*‐Cre mice and *Cd38* ^fl/fl^ littermate controls with KP. *Lyz2*‐Cre‐mediated *Cd38* deletion improved survival after KP infection, whereas 78C treatment did not provide an obvious additional survival benefit in *Cd38* ^fl/fl^ *Lyz2*‐Cre mice (Figure 8A). Consistently, *Cd38* ^fl/fl^ *Lyz2*‐Cre mice showed reduced clinical sepsis scores compared with infected *Cd38* ^fl/fl^ controls (Figure 8B). *Lyz2*‐Cre‐mediated *Cd38* deletion also reduced KP burden in the lung and liver and showed a similar downward trend in the spleen (Figure 8C). Histological analysis further showed that *Cd38* ^fl/fl^ *Lyz2*‐Cre mice were protected from KP‐induced inflammatory lung injury, with reduced alveolar inflammation, hemorrhagic consolidation, and lung injury scores (Figure 8D,E). We then performed a gentamicin‐protection assay using BMDMs from *Cd38* ^fl/fl^ and *Cd38* ^fl/fl^ *Lyz2*‐Cre mice (Figure 8F). *Cd38*‐deficient BMDMs showed a more pronounced reduction at later time points (Figure 8G,H). These data indicate that *Cd38* deficiency enhances intracellular KP clearance in macrophages in vitro. Together, these findings support a functional contribution of myeloid CD38 to KP‐induced sepsis, bacterial dissemination, and tissue injury.

**FIGURE 8:**
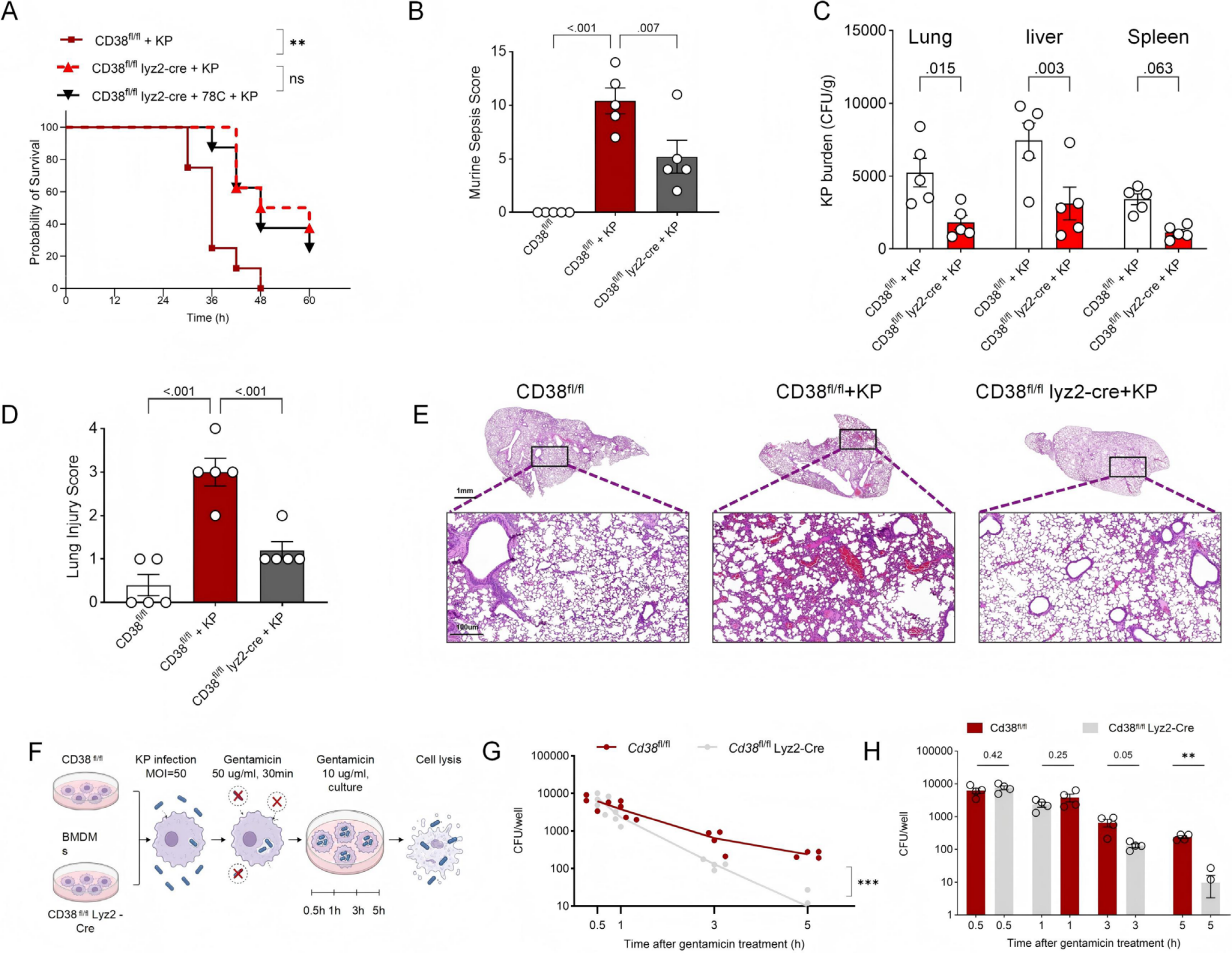
Myeloid *Cd38* Deletion Attenuates KP‐induced Sepsis. (A) Kaplan–Meier survival curves of KP‐infected *Cd38* ^fl/fl^ mice (*n* = 8), *Cd38* ^fl/fl^ *Lyz2*‐Cre mice (*n* = 8) and 78C‐treated *Cd38* ^fl/fl^ *Lyz2*‐Cre mice (*n* = 8). Survival was monitored for 60 h after KP infection. Statistical significance was assessed by the log‐rank test. (B) Murine sepsis scores in *Cd38* ^fl/fl^, KP‐infected *Cd38* ^fl/fl,^ and KP‐infected *Cd38* ^fl/fl^ *Lyz2*‐Cre mice. (C) KP bacterial burden in lung, liver, and spleen from KP‐infected *Cd38* ^fl/fl^ and *Cd38* ^fl/fl^ *Lyz2*‐Cre mice, quantified as CFU per gram of tissue. (D) Quantification of lung injury scores in *Cd38* ^fl/fl^, KP‐infected *Cd38* ^fl/fl,^ and KP‐infected *Cd38* ^fl/fl^ *Lyz2*‐Cre mice. (E) Representative H&E‐stained lung sections from *Cd38* ^fl/fl^, KP‐infected *Cd38* ^fl/fl,^ and KP‐infected *Cd38* ^fl/fl^ *Lyz2*‐Cre mice. (F) Schematic of the gentamicin‐protection assay for quantifying viable intracellular KP in BMDMs. (G, H) Time‐course analysis (G) and quantification at the indicated time points (H) of intracellular KP burden in BMDMs from *Cd38* ^fl/fl^ and *Cd38* ^fl/fl^ *Lyz2*‐Cre mice after gentamicin treatment. Statistical analysis was performed using two‐way ANOVA with genotype and time as factors, including the genotype × time interaction, followed by Sidak‐adjusted comparisons between genotypes at individual time points.

## 3 Discussion

In this study, we applied scH16S‐seq to link bacterial 16S‐derived UMI signals with host transcriptional states at single‐cell resolution during KP‐induced sepsis. This approach identified *Cd38* ^+^ *Acod1* ^+^ monocyte‐derived macrophages, *Cd38* ^+^ *Lyve1* ^+^ interstitial macrophages, and *Cd38* ^+^ hypoxic neutrophils as myeloid cell states with high KP UMI loads. Functionally, CD38‐targeted and NAD‐related interventions reduced KP‐induced inflammatory activation, preserved mitochondrial–lysosomal fitness, and promoted intracellular KP clearance.

The first implication of this work is methodological. By integrating bacterial 16S‐targeted signal recovery with host transcriptome profiling under a shared single‐cell barcode, scH16S‐seq provides a practical framework to ask not only “which cells are inflamed”, but “which inflamed cells are associated with bacterial signal”. This distinction is important because infection‐induced transcriptional activation is widespread, whereas bacterial UMI enrichment was concentrated in discrete myeloid states.

Our results further characterize bacterial signal‐enriched myeloid states in KP‐induced sepsis. KP is often regarded as an extracellular pathogen, yet previous studies have shown that it can survive within macrophages by altering phagosome maturation and avoiding efficient delivery to lysosomes [4, 29]. In vivo single‐cell work further identified lung interstitial macrophages as a major macrophage population associated with KP and showed that KP can skew macrophage polarization to promote infection [5]. Our scH16S‐seq data are consistent with this concept by identifying macrophage and neutrophil states enriched for KP 16S‐derived signals.

CD38 emerged as a shared marker across these bacterial UMI‐enriched myeloid states. CD38 has long been recognized as a multifunctional immune ectoenzyme that can increase NADase activity, reduce NAD^+^ availability, and reshape macrophage metabolism [25, 28, 30]. CD38 was increased in monocytes across CITE‐seq, sorted CD14^+^ monocyte, and single‐cell sepsis datasets, consistent with recent reports that CD38high monocytes distinguish bacterial sepsis and associate with adverse outcomes [15, 23]. Thus, our work links a clinically observed CD38^high^ myeloid phenotype to cell‐resolved bacterial signal‐enriched states and an organelle dysfunction model that is functionally supported by pharmacological and genetic perturbation. Emerging evidence indicates that NAD metabolism is also a key determinant of host responses to infection and sepsis [31, 32, 33]. Our mechanistic analyses further position mitochondrial bioenergetics between CD38–NAD dysregulation and lysosomal antibacterial function. KP infection reduced NAD^+^ availability, the NAD^+^/NADH ratio, cellular ATP, and mitochondrial respiratory capacity, whereas CD38 inhibition, NAM treatment, and *Cd38* deficiency restored these parameters to varying degrees. Importantly, inhibition of mitochondrial ATP synthesis with oligomycin or V‐ATPase function with bafilomycin A1 attenuated the recovery of both lysosomal acidification and intracellular KP clearance. These pharmacological dependency experiments support a model in which the antibacterial benefit of CD38–NAD modulation depends, at least in part, on restoration of mitochondrial energy production and intact V‐ATPase‐dependent lysosomal acidification.

Host‐directed therapy aims to restore protective immunity while limiting tissue‐damaging inflammation [34]. Our data fit this concept. CD38‐targeted and NAD‐related interventions reduced inflammatory signatures without compromising bacterial control; instead, they improved intracellular KP killing and reduced tissue bacterial burden in vivo. In this setting, dampening maladaptive inflammatory metabolism can improve, rather than impair, pathogen clearance. Future work should also test whether this axis generalizes beyond KP. Our supportive PA analysis suggests that *Cd38* ^+^ inflammatory myeloid states may also be enriched for bacterial 16S‐derived UMI signals in another Gram‐negative infection setting. More broadly, scH16S‐seq may provide a generalizable framework for cell‐resolved investigation of host responses to other clinically important bacterial pathogens. With appropriate organism‐specific validation of 16S capture and sample‐processing conditions, this approach could potentially be extended to infections caused by *Staphylococcus aureus*, *Mycobacterium tuberculosis*, and other bacterial species, enabling identification of host cell states preferentially associated with bacterial 16S‐derived signals across diverse infectious settings. However, whether CD38 functionally regulates antibacterial defense in PA or other pathogens remains to be tested.

An important next step will be to integrate cell‐resolved molecular profiling with intravital imaging to resolve the temporal dimension of host–bacterial interactions in vivo. Recent advances in confocal scanning light‐field microscopy, computational adaptive optics, high‐numerical‐aperture volumetric imaging, and long‐term focal stabilization have substantially expanded the duration, fidelity, field of view, and stability of three‐dimensional intravital imaging in living tissues, enabling prolonged visualization of dynamic immune‐cell behaviors, including neutrophil migration and neutrophil–macrophage interactions [35, 36, 37, 38]. Moreover, studies integrating intravital imaging with single‐cell and spatial transcriptomic analyses have demonstrated the value of linking dynamic myeloid‐cell behavior with molecularly defined cell states in pathological tissue microenvironments [39, 40]. Integration of these approaches with scH16S‐seq could directly determine the duration and fate of bacterial contact, uptake, retention, and clearance by defined *CD38* ^+^ myeloid populations and establish how these dynamics are altered by CD38‐targeted intervention or myeloid *CD38* deletion.

Several limitations should be considered. Bacterial 16S‐derived UMI burden reflects cell‐associated bacterial signal, not necessarily viable intracellular bacteria. Although control lungs showed low baseline KP 16S‐like signals and infection‐associated signals were preferentially enriched in phagocytic myeloid compartments, low‐level environmental or extracellular bacterial nucleic‐acid contributions cannot be completely excluded. Second, because *Lyz2*‐Cre targets multiple myeloid lineages, including monocytes/macrophages and neutrophils, the protective phenotype observed in *Cd38* ^fl/fl^ *Lyz2*‐Cre mice should be interpreted as evidence for a general myeloid contribution of CD38. Future studies using lineage‐selective *Cd38* deletion, bone‐marrow chimeras, or selective depletion approaches will be required to resolve the relative contributions of macrophage and neutrophil populations. Third, human PBMC datasets support the clinical relevance of CD38 upregulation in sepsis‐associated myeloid cells, but they do not directly demonstrate bacterial signal‐enriched lung myeloid states during human sepsis.

In summary, our study provides a cell‐resolved view of KP‐induced sepsis by linking bacterial 16S‐derived signals to host myeloid states and antibacterial function. We identify *Cd38* ^+^ macrophage and neutrophil populations as bacterial 16S UMI‐enriched inflammatory myeloid states and show that CD38‐targeted and NAD‐related interventions preserve mitochondrial–lysosomal fitness, promote intracellular KP clearance, and improve disease outcomes.

## 4 Methods

### 4.1 Animals

C57BL/6 mice aged 6–8 weeks were obtained from GemPharmatech LLC. *Cd38* ^fl/fl^ mice and myeloid‐specific *Cd38* knockout mice (*Cd38* ^fl/fl^ *Lyz2*‐Cre mice with *Lyz2*‐Cre‐mediated *Cd38* deletion across myeloid lineages) were kindly provided by Professor Hongbo Xin of Nanchang University and maintained under specific pathogen‐free conditions at 20°C–24°C under a 12‐h light/dark cycle, with free access to food and water [41]. All animal procedures were approved by the Institutional Animal Care and Use Committee of Jinling Hospital (approval no. DZYISKTB20240317001) and performed in accordance with national guidelines for the humane care and use of laboratory animals. Public human datasets were analyzed in de‐identified form.

### 4.2 Bacterial Strains and Culture

*Klebsiella pneumoniae* NTUH‐K2044 and *Pseudomonas aeruginosa* PA14 were cultured in Luria–Bertani broth at 37°C with shaking at 300 rpm and harvested during exponential growth. Bacterial density was estimated by optical density at 600 nm and confirmed by colony‐forming unit (CFU) enumeration on LB agar plates. Before infection, bacteria were washed three times with sterile PBS and resuspended at the indicated concentrations.

### 4.3 In Vivo Experiments

For survival analysis, mice were infected intraperitoneally with 1 × 10^3^ CFU KP NTUH‐K2044 and treated 1 h later with vehicle, CD38 inhibitor 78C (15 mg/kg; MedChemExpress, HY‐123999) or nicotinamide (NAM; 200 mg/kg; MedChemExpress, HY‐B0150). All interventions were given as a single intraperitoneal injection, and survival was monitored for 60 h.

For tissue analysis, mice were infected intraperitoneally with 2 × 10^3^ CFU KP and treated with vehicle, 78C, or NAM as described above. Sepsis severity was assessed 24 h after infection using a predefined murine sepsis scoring system based on general appearance, activity, posture, response to stimulation, and respiratory status, with each parameter scored from 0 to 3. Intestine, lung, spleen, and liver tissues were collected 24 h after infection for histology, bacterial burden measurement, and lung qPCR analysis. Mice were randomly assigned to experimental groups, and histological injury scoring was performed in a blinded manner.

### 4.4 Mouse Infection Models for scH16S‐seq

For scH16S‐seq analysis, mice were assigned to four groups: Control, KP non‐septic infection (KP_NS), KP septic infection (KP_S), and PA septic infection (PA_S). Control mice received sterile PBS intraperitoneally. KP_NS mice were infected intraperitoneally with 2 × 10^3^ CFU KP and collected 12 h after infection. KP_S mice were infected intraperitoneally with 2 × 10^3^ CFU KP and collected 24 h after infection. PA_S mice were infected intraperitoneally with 2 × 10^4^ CFU P. aeruginosa and collected 24 h after infection. Whole lung tissues were harvested for single‐cell suspension preparation and host‐bacterial single‐cell profiling.

### 4.5 Single‐Cell Host Transcriptome and Bacterial 16S Co‐Sequencing

The scH16S‐seq analysis was designed primarily for cell‐type‐ and cell‐state‐resolved mapping of cell‐associated bacterial 16S‐derived signals. For each condition, lungs from three mice were pooled before SCOPE‐chip capture, and each pooled sample was processed as a separate library and sequenced independently. Because pooling preceded single‐cell barcoding, animal‐of‐origin information was not retained, and the contributing mice did not constitute independent scH16S‐seq replicates. Accordingly, no animal‐level pseudobulk or mixed‐effects analysis of between‐condition differences was performed, and individual cells were not treated as independent biological replicates. Bacterial‐signal‐positive fractions and UMI burdens were summarized descriptively across annotated cell types and transcriptional states within each pooled dataset to identify populations preferentially associated with higher bacterial‐signal burden.

### 4.6 Primary Processing of Host Transcriptome and Bacterial 16S Libraries

Host transcriptome reads were processed using CeleScope v2.0.6 with default parameters. After demultiplexing, reads were aligned to the mouse reference genome mm39/GRCm39 using STAR v2.6.1a, assigned to exonic features with featureCounts v2.0.1, and collapsed by cell barcode, UMI, and gene assignment to generate gene‐by‐cell UMI matrices.

Bacterial 16S libraries were processed using CeleScope v2.0.6 and aligned separately with STAR v2.6.1a to the Klebsiella pneumoniae NTUH‐K2044 (NC_012731.1) and Pseudomonas aeruginosa PA14 (NC_008463.1) reference sequences using –outFilterMatchNmin 80. Alignment metrics were recorded before feature assignment. Reads mapping uniquely within each reference alignment were assigned to bacterial 16S rRNA features using featureCounts v2.0.1. A UMI‐support filtering strategy adapted from Viral‐Track was applied, retaining bacterial UMIs supported by at least three reads. Filtered KP‐ and PA‐reference‐aligned UMI tables were matched to host cell barcodes retained after quality control and imported into the host Seurat object.

KP‐ or PA‐reference‐positive cells were defined as cells containing at least one corresponding bacterial 16S‐derived UMI after filtering and barcode matching. Within each pooled dataset, positive‐cell fractions were calculated relative to all retained cells in the corresponding cell type or state. Mean bacterial UMI counts were calculated across all retained cells for Table S2 and only among cells positive for the corresponding bacterial 16S‐derived signal for the bubble plots in Figures 2F and 3F, and Figures S2C and S3D.

### 4.7 Host Single‐Cell Transcriptome Analysis

Downstream analysis was performed in R v4.5.0 using Seurat v5.5.0 and SeuratObject v5.4.0. Samples were labeled as Control, KP_NS, KP_S, and PA_S, corresponding to control, KP non‐septic infection, KP septic infection, and PA septic infection supportive comparison samples, respectively. Seurat objects were generated with `min.cells = 3` and `min.features = 200`. Cells were retained if they met the following criteria: nCount_RNA ≥ 500, nFeature_RNA ≥ 200, nFeature_RNA ≤ 6000, and mitochondrial transcript percentage ≤ 10%. After filtering, 74 960 lung cells were retained, including 19 294 Control cells, 22 002 KP_NS cells, 15 884 KP_S cells, and 17 780 PA_S cells.

Filtered objects were normalized using SCTransform with mitochondrial percentage regressed out, followed by principal component analysis. Datasets were integrated using Harmony v1.2.4 with sample_id as the integration variable. UMAP embedding and graph‐based clustering were performed using Harmony dimensions 1–32, with a clustering resolution of 0.6 used for final global annotation. Cluster markers were identified using FindAllMarkers on the SCT assay with positive markers only, min.pct = 0.25, and logfc.threshold = 0.25, to support cell‐state annotation and marker prioritization within the integrated dataset; these cell‐level marker analyses were not used to support formal between‐condition biological inference.

### 4.8 Reclustering of Myeloid Compartments

Neutrophils were subsetted from the globally annotated lung object and reclustered independently. The subset was re‐normalized using SCTransform with mitochondrial percentage regressed out, followed by PCA, Harmony integration, and UMAP embedding. Putative contaminant clusters were removed based on marker expression and QC inspection, and retained neutrophils were reprocessed using the same workflow before final state annotation.

For mononuclear phagocyte analysis, alveolar macrophages and non‐alveolar mononuclear phagocytes were subsetted from the global object. The subset was re‐normalized using SCTransform with mitochondrial percentage regressed out, followed by PCA, Harmony integration, UMAP embedding, and graph‐based clustering. Fine‐state annotations were assigned based on canonical marker expression and differentially expressed genes.

Functional and metabolic gene modules were scored at the single‐cell level using `AddModuleScore` in Seurat.

### 4.9 Public Human Sepsis Dataset Analysis

Human validation was performed using publicly available GEO datasets. Adult CITE‐seq data from GSE279451 were used to assess concordant RNA and surface‐protein changes between patients with sepsis and healthy controls. Donor‐level pseudobulk RNA and antibody‐derived tag (ADT) profiles were generated for 32 patients with sepsis and three healthy controls. Purified peripheral‐blood CD14^+^ monocyte datasets GSE133822, GSE139913, and GSE11755 were analyzed using healthy‐control and sepsis samples only. Adult PBMC scRNA‐seq data from GSE279452 were analyzed for cell‐state‐level validation and included healthy controls (*n* = 7), ICU controls without sepsis (*n* = 8) and patients with sepsis (*n* = 93). PBMC profiles were integrated using Harmony, annotated into major immune compartments, and reclustered within the myeloid compartment to define 11 myeloid states.

### 4.10 Cell Culture and Macrophage Infection

iBMDMs and RAW264.7 cells were cultured in Dulbecco's modified Eagle's medium (DMEM; G4512, Servicebio) supplemented with 10% fetal bovine serum (FBS; 10099141, Gibco), 100 U/mL penicillin and 100 µg/mL streptomycin (15140122, Gibco) at 37°C in 5% CO_2_. THP‐1 cells were cultured in RPMI 1640 medium supplemented with 10% FBS and penicillin–streptomycin, and were differentiated with phorbol 12‐myristate 13‐acetate (PMA; 10 ng/mL) for 24 h before infection.

Primary bone marrow‐derived macrophages (BMDMs) were generated from femurs and tibias of 6–8‐week‐old mice. Bone marrow cells were flushed, subjected to red blood cell lysis (C3702, Beyotime), filtered, and cultured in DMEM containing 10% FBS, penicillin–streptomycin and 20 ng/mL macrophage colony‐stimulating factor (M‐CSF; PeproTech). The medium was replaced every 3 days, and mature BMDMs were used on day 7.

For in vitro infection, macrophages were seeded at 5 × 10^5^ cells per well in 12‐well plates or 1 × 10^6^ cells per well in 6‐well plates. Before infection, the culture medium was replaced with fresh antibiotic‐free medium. Cells were infected with live *Klebsiella pneumoniae* NTUH‐K2044 at an MOI of 50 for 3 h unless otherwise indicated. Where indicated, cells were pretreated with 78C (2 µM, 1 h) or nicotinamide (NAM; 2 mM, 4 h) before KP infection; control cells received vehicle. After infection, extracellular bacteria were reduced by washing cells three times with PBS. For imaging assays, cells were processed directly after washing. For flow cytometry, RNA extraction, and protein analysis, cells were detached with EDTA, collected by centrifugation at 600 × g for 3 min, and processed immediately.

### 4.11 Immunofluorescence Staining and Fluorescence in Situ Hybridization

For cultured‐cell immunofluorescence, cells were washed after infection, fixed with 4% paraformaldehyde, permeabilized with 0.1% Triton X‐100 and blocked with 5% BSA. For paraffin‐embedded tissue sections, sections were deparaffinized, rehydrated, and subjected to citrate‐buffer antigen retrieval, followed by permeabilization and blocking. Cells or tissue sections were incubated with primary antibodies overnight at 4°C, followed by fluorophore‐conjugated secondary antibodies and nuclear counterstaining. For these assays, CD38 was detected using an anti‐CD38 monoclonal antibody (60006‐1‐Ig, Proteintech), and KP was detected using an anti‐Klebsiella pneumoniae antibody (ab20947, Abcam).

For combined KP 16S fluorescence in situ hybridization (FISH) and immunofluorescence, lung sections were stained using KP 16S‐FISH reagents (GW16S01, TinyGen) together with a rat anti‐mouse CD38 monoclonal antibody and a rabbit anti‐CD11b antibody. Nuclei were counterstained with DAPI. The proportions of CD38^+^ and CD38^−^ cells among KP‐FISH^+^CD11b^+^ myeloid cells were quantified. Cell counts from multiple imaging fields were pooled within each mouse before calculating these proportions, and individual mice were considered independent biological replicates.

### 4.12 Western Blotting

Protein lysates were prepared from mouse lung tissue collected 24 h after KP infection and from BMDMs collected at the indicated time points after KP infection (MOI 50). Equal amounts of protein were separated by SDS–PAGE and transferred to PVDF membranes. Membranes were incubated with anti‐CD38 antibody (YA6174, MedChemExpress, HY‐P86482) or anti‐GAPDH antibody (GB15002‐100, Servicebio), followed by the corresponding HRP‐conjugated secondary antibodies. Protein bands were visualized by chemiluminescence, and CD38 abundance was quantified by densitometry and normalized to GAPDH where indicated.

### 4.13 Flow Cytometry and Mitochondrial Assays

For CD38 flow cytometry, infected iBMDMs were washed, detached with EDTA, and incubated with anti‐CD38 antibody (60006‐1‐Ig, Proteintech), followed by an APC‐conjugated secondary antibody. Cells and singlets were gated before quantification of CD38 fluorescence using an ABCyte C3140 flow cytometer.

Mitochondrial superoxide was measured in live iBMDMs using a MitoSOX Red mitochondrial superoxide indicator kit (S0061M, Beyotime). After KP infection with or without 78C or NAM pretreatment, cells were incubated with MitoSOX Red working solution for 15 min at 37°C, protected from light, washed, and analyzed immediately by flow cytometry or live‐cell fluorescence imaging.

Mitochondrial membrane potential was assessed using a JC‐1 mitochondrial membrane potential assay kit (C2006, Beyotime). Cells were incubated with JC‐1 working solution for 20 min at 37°C, washed with JC‐1 staining buffer, and analyzed by flow cytometry or fluorescence imaging. Mitochondrial depolarization was quantified by increased JC‐1 green monomer signal and reduced JC‐1 red aggregate signal or red/green fluorescence balance.

### 4.14 RNA Extraction and Quantitative PCR

Total RNA was extracted from cultured cells or mouse tissues using TRIzol reagent (Invitrogen). RNA was reverse‐transcribed using HiScript III RT SuperMix (R323, Vazyme), and quantitative PCR was performed on a QuantStudio 3 Real‐Time PCR System (Applied Biosystems) using SYBR Green qPCR Master Mix (Q711, Vazyme). Relative gene expression was normalized to ACTB and calculated using the 2^‐ΔΔCt method. Mouse *Ifnb1*, *Il6*, *Il1b*, *Cd38*, *Cox1*, *Cox2*, and *Cox3*, as well as human *CD38*, *IL6*, and *IFNB1*, were quantified by qPCR. Primer sequences are provided in Table S3.

### 4.15 Bulk RNA‐seq Analysis

iBMDMs were assigned to Control, KP, KP+78C, and KP+NAM groups, with three biological replicates per group. Gene count, normalized count, and transcripts per million (TPM) matrices were generated by the sequencing analysis pipeline.

Differential‐expression analysis was performed in R using DESeq2. Genes were retained if raw counts were ≥10 in at least three samples. The design formula was set as group. Variance‐stabilized transformed counts were used for principal component analysis, sample‐distance heatmaps, and correlation heatmaps.

### 4.16 MitoTracker and LysoTracker Live‐Cell Imaging

To assess mitochondrial–lysosomal fitness, iBMDMs were infected with live KP at MOI 50 for 3 h, with or without 78C or NAM pretreatment. Cells were stained with MitoTracker Red CMXRos (50 nM), LysoTracker Green DND‐26 (50 nM), and Hoechst for 15 min before live‐cell imaging. CCCP (10 µM, 30 min) was used as a control for mitochondrial depolarization and lysosomal deacidification.

### 4.17 Ratiometric Lysosomal pH Assay

Lysosomal acidification was assessed using LysoSensor Yellow/Blue DND‐160. iBMDMs were infected with live KP at MOI 50 for 3 h with the indicated 78C or NAM pretreatment, stained with LysoSensor Yellow/Blue DND‐160 (1 µM, 5 min), and analyzed using a plate reader. The 540/440 nm fluorescence ratio was used as an index of lysosomal acidification, with higher values indicating greater acidification. CCCP was included as a deacidification control. Where indicated, cells were additionally treated with oligomycin (2 µM, 3 h) or bafilomycin A1 (100 nM, 3 h) to inhibit mitochondrial ATP synthase or V‐ATPase, respectively, before LysoSensor measurement.

### 4.18 Gentamicin‐Protection Intracellular Bacterial Survival Assay

Intracellular KP survival was assessed using a gentamicin‐protection assay. Macrophages were infected with live KP at MOI 50 for the indicated duration. Extracellular bacteria were eliminated with gentamicin (50 µg/mL, 30 min), followed by culture in medium containing gentamicin (10 µg/mL). At the indicated time points, cells were lysed with 0.1% Triton X‐100, and viable intracellular KP was quantified by serial dilution and CFU plating. For time‐resolved analysis of intracellular KP persistence and clearance, the end of the high‐dose gentamicin step was designated T0; cells were subsequently collected at the indicated time points for parallel anti‐KP immunofluorescence and intracellular CFU quantification. For pharmacological dependency experiments, KP‐infected macrophages treated with 78C or NAM were additionally exposed to oligomycin or bafilomycin A1 under the indicated conditions, and viable intracellular KP was quantified 4 h after gentamicin protection.

### 4.19 Histology and Tissue Bacterial Burden

For histological analysis, intestine, lung and spleen tissues were collected 24 h after KP infection, fixed in 4% paraformaldehyde, embedded in paraffin, sectioned and stained with hematoxylin and eosin. Tissue injury scores for intestine, lung and spleen were assigned according to predefined histopathological criteria.

For bacterial burden analysis in the intervention experiments, liver, lung, and spleen tissues were collected 24 h after KP infection. For characterization of the scH16S‐seq infection cohorts, lung tissues were collected at 12 h from KP_NS mice and 24 h from KP_S mice. Tissues were weighed and homogenized in sterile PBS. Homogenates were serially diluted, plated on LB agar, and incubated at 37°C for more than 12 h. Bacterial loads were expressed as CFU per gram of tissue.

### 4.20 Intracellular NAD^+^/NADH and ATP Measurements

Intracellular NAD^+^ and NADH levels were measured using an NAD^+^/NADH Assay Kit (WST‐8; HY‐K0313, MedChemExpress) according to the manufacturer's instructions. Briefly, cells were harvested and lysed in pre‐chilled extraction buffer, followed by centrifugation at 12 000 × g at 4°C to collect the supernatant. Total NAD(H) and NADH were quantified using the WST‐8‐based colorimetric assay, with absorbance measured at 450 nm. For selective determination of NADH, aliquots of the extracts were heated at 60°C for 30 min to decompose NAD^+^. NAD^+^ abundance was calculated by subtracting NADH from total NAD(H), and the NAD^+^/NADH ratio was calculated accordingly.

Intracellular ATP levels were determined using an ATP Assay Kit (HY‐K0314, MedChemExpress) according to the manufacturer's protocol. Cell lysates were clarified by centrifugation at 12 000 × g at 4°C, and ATP‐dependent luminescence was measured using the luciferin–luciferase reaction on a microplate reader. ATP levels were calculated from an ATP standard curve. NAD^+^, NADH, and ATP abundance were normalized to cell number and expressed as pmol per 10^5^ cells.

### 4.21 Seahorse Mitochondrial Stress Test

Mitochondrial respiration was assessed using a Seahorse XF96 Extracellular Flux Analyzer (Agilent Technologies) with the XF Cell Mito Stress Test Kit. Cells were plated in XF96 microplates at a density of 5 × 10^4^ cells per well, subjected to the indicated treatments, and infected with KP for 3 h. Before measurement, the culture medium was replaced with Seahorse XF DMEM containing 10 mM glucose, 2 mM glutamine, and 1 mM pyruvate, and the cells were equilibrated for 1 h at 37°C in a non‐CO_2_ incubator. Oxygen consumption rate (OCR) was monitored at baseline and following sequential addition of oligomycin (1.5 µM), FCCP (1.5 µM), and rotenone plus antimycin A (0.5 µM each). Respiratory parameters, including basal respiration, ATP‐linked respiration, maximal respiration, and non‐mitochondrial oxygen consumption, were derived using Wave software. OCR measurements were normalized to the number of cells per well, which was determined in parallel wells under identical experimental conditions using a Countess II FL automated cell counter (Thermo Fisher Scientific).

### 4.22 Statistical Analysis

Statistical analyses were performed using GraphPad Prism 9 unless otherwise indicated. Data are presented as mean ± SEM, with individual data points representing independent biological replicates. Individual mice were considered biological replicates for in vivo experiments, whereas independent experiments or cell preparations were considered biological replicates for in vitro studies. Comparisons between two independent groups were performed using an unpaired two‐tailed Student's t‐test or Welch's t‐test when variances were unequal. Comparisons among multiple groups were performed using one‐way ANOVA followed by Sidak‐adjusted multiple comparisons where appropriate. Kaplan–Meier survival curves were compared using the log‐rank (Mantel–Cox) test. Gentamicin‐protection time‐course data were analyzed using ordinary two‐way ANOVA with treatment/genotype and time as factors, including the treatment/genotype × time interaction, followed by Sidak‐adjusted comparisons between groups at individual time points. Statistical significance was defined as *p* < 0.05.

## Author Contributions

Conceptualization, Y.G.L., Y.Z., J.A.R. and X.W.W.; Methodology, Y.G.L., Y.L.Y. and F.Q.; Investigation, Y.G.L., S.X.L., X.H.L., P.Z.L., J.H.L., H.Q.L., S.Q.J., J.H.Z, J.Y.L., S.C.L., and T.Z.; Formal analysis, Y.G.L. and F.Q.; Data curation, Y.G.L. and F.Q.; Visualization, Y.G.L.; Funding acquisition, Y.L.Y., Y.Z., J.A.R., and X.W.W.; Supervision, Y.Z., J.A.R., and X.W.W.; Writing – original draft, Y.G.L.; Writing – review & editing, Y.G.L., Y.L.Y., Y.Z., J.A.R., and X.W.W. All authors discussed the results and approved the final manuscript.

## Conflicts of Interest

F.Q. is an employee of Singleron Biotechnologies, which provides the FocuSCOPE 16S workflow used in this study. F.Q. provided technical support related to scH16S‐seq library preparation, sequencing, and primary data processing. The remaining authors declare no conflicts of interest.

## Supporting information




**Supporting File**: advs78127‐sup‐0001‐SuppMat.docx.

## Data Availability

The raw scH16S‐seq sequencing data generated in this study have been deposited in the NCBI under BioProject accession PRJNA1509723. Additional data that support the findings of this study are available on request from the corresponding author.
