# Supplementary material for: scH16S‐Seq Maps Klebsiella pneumoniae‐Associated Myeloid States and Reveals a CD38–NAD Immunometabolic Axis That Impairs Lysosomal Acidification in Sepsis
Source: Adv Sci (Weinh). 2026 Sep 30:e78127. Online ahead of print. doi: 10.1002/advs.78127 (PMC13626163; doi:10.1002/advs.78127)
Supplement: Supplementary file 1 [file ADVS-9999-e78127-s001.docx]

**Supporting Information**

## Supplementary Figures

## Figure S1 | Quality control, global cell composition and supportive PA analysis of bacterial 16S UMI mapping.

(A) Distribution of single-cell quality-control metrics across Control, KP_NS, KP_S, and PA_S scH16S-seq datasets, including the number of detected genes per cell, total UMI counts per cell, and the percentage of mitochondrial transcripts.

(B) Histological lung injury scores in Control, KP_NS, and KP_S mice.

(C) Quality-control distributions of detected genes, total RNA UMI counts and mitochondrial transcript percentage across samples.

(D) UMAP embedding of integrated lung cells colored by sample origin.

(E) Global proportions and absolute numbers of major cell types across Control, KP_NS, KP_S and PA_S groups.

(F) Load-stratified frequencies of PA 16S UMI-positive cells across major lung cell types in Control and PA_S groups.

(G) Distribution of PA-associated 16S-derived UMI signals across major lung cell types.

(H) Technical-control analysis showing correlations between log1p-transformed PA 16S-derived UMI counts and log10-transformed host nCount_RNA or nFeature_RNA. Spearman correlation coefficients are shown.

## Figure S2 | Supportive PA analysis and QC assessment of bacterial 16S UMI-enriched macrophage states.

(A) UMAP of the reclustered mononuclear phagocyte compartment colored by Control and PA_S sample groups.

(B) Load-stratified frequencies of PA 16S UMI-positive cells across fine mononuclear phagocyte states.

(C) Bubble plot showing PA 16S-derived UMI enrichment across fine mononuclear phagocyte states. Bubble size indicates the PA 16S UMI-positive rate, and color indicates the mean PA 16S-derived UMI count among positive cells.

(D) Quality-control metric distributions across fine mononuclear phagocyte states, including detected genes per cell, total RNA UMI counts and mitochondrial transcript percentage.

## Figure S3 | Supportive PA analysis of bacterial 16S UMI-enriched CD38⁺ hypoxic neutrophils.

(A) Heatmap showing Gene Ontology enrichment results for marker genes from CD38⁺ hypoxic neutrophils, CD38⁺ Acod1⁺ mo-Macs and CD38⁺ Lyve1⁺ IMs. Marker genes were defined by |log2FC| > 0.5.

(B) UMAP of reclustered neutrophils colored by Control and PA_S sample groups.

(C) Load-stratified frequencies of PA 16S UMI-positive cells across neutrophil states.

(D) Bubble plot showing PA 16S-derived UMI enrichment across neutrophil states. Bubble size indicates the PA 16S UMI-positive rate, and color indicates the mean PA 16S-derived UMI count among positive cells.

(E) Quality-control metric distributions across neutrophil states, including detected genes per cell, total RNA UMI counts and mitochondrial transcript percentage.

(F) Representative multiplex imaging of lung sections from Control and KP-infected mice using KP 16S-FISH together with CD38 and the myeloid marker CD11b. DAPI is shown in blue, KP 16S-FISH in green, CD11b in red, and CD38 in yellow.

(G) Quantification of CD38⁺ and CD38⁻ fractions among KP-FISH⁺CD11b⁺ myeloid cells in KP-infected lung tissue. Multiple imaging fields were aggregated within each mouse, and each point represents one individual mouse (n = 3 mice).

## Figure S4 | KP induces CD38 expression, while CD38/NAD-related interventions attenuate myeloid inflammatory activation.

(A) Representative immunofluorescence images showing Hoechst, CD38, and KP signals in iBMDMs infected with live KP for 3 h at MOI 50, with or without 78C pretreatment (2 μM, 1 h) or NAM pretreatment (2 mM, 4 h). Boxed regions are shown at higher magnification. Scale bars, 20 μm; enlarged images, 5 μm.

(B) Quantification of CD38 mean fluorescence intensity (MFI) in the groups shown in A.

(C) Representative immunoblot of CD38 and GAPDH in lung tissue from Control and KP-infected mice collected 24 h after infection.

(D) Densitometric quantification of CD38 protein abundance normalized to GAPDH in the groups shown in C. n = 3 mice per group.

(E) Representative immunoblot showing CD38 protein expression in BMDMs collected at 0, 1, and 3 h after KP infection at MOI 50. GAPDH was used as a loading control.

(F) Representative flow-cytometry gating strategy for cell and singlet selection and CD38-APC fluorescence histograms in iBMDMs infected with live KP for 3 h at MOI 50, with or without 78C or NAM pretreatment.

(G) Heatmap of representative inflammatory, chemokine, and interferon-related genes in iBMDMs across Control, KP, KP + 78C, and KP + NAM groups.

(H) Relative *Il1b*, *Ifnb1*, and *Il6* mRNA expression in primary BMDMs infected with live KP for 3 h at MOI 50, with or without 78C or NAM pretreatment. Expression was normalized to Actb.

(I) Relative *Cd38* mRNA expression in PMA-differentiated THP-1 cells with or without KP infection. Expression was normalized to *Actb*.

(J) Relative *IL6* and *IFNB1* mRNA expression in PMA-differentiated THP-1 cells across Control, KP, KP + 78C, and KP + NAM groups. Expression was normalized to *Actb*.

## Figure S5 | KP infection impairs mitochondrial function and lysosomal acidification.

(A) Heatmap of mitochondrial respiratory-chain and complex IV-associated genes in iBMDMs after live KP infection for 3 h at MOI 50. Log2 fold changes and adjusted P values are shown.

(B) Relative Cox1, Cox2 and Cox3 mRNA expression in lung tissue collected 24 h after KP infection. Where indicated, mice received 78C (15 mg/kg) or NAM (200 mg/kg) 1 h after KP infection. qPCR data were normalized to Actb.

(C) Representative live-cell fluorescence images of MitoTracker Red CMXRos, LysoSensor Green DND-26 and Hoechst in iBMDMs treated with CCCP (10 μM, 30 min) or vehicle control.

(D) Quantification of normalized CMXRos and LysoTracker Green DND-26 fluorescence intensity in CCCP-treated and control iBMDMs.

(E) Proposed model. KP infection is associated with mitochondrial depolarization, impaired lysosomal acidification and reduced organelle-dependent antibacterial fitness. CD38 inhibition or NAM treatment partially preserves mitochondrial–lysosomal function and enhances intracellular KP clearance.

## Supplementary Tables

## Table S1.Distribution of bacterial 16S-derived signals across called-cell and background barcodes in control and septic infection datasets.

| Sample | Barcode class | N barcodes^c)^ | KP+ barcodes (n) | KP+ rate (%)^d)^ | KP UMI sum | KP UMI density (/1,000 barcodes)^e)^ | Mean KP UMI per KP+ barcode | PA+ barcodes (n) | PA+ rate (%)^d)^ | PA UMI sum | PA UMI density (/1,000 barcodes)^e)^ | Mean PA UMI per PA+ barcode |
| --- | --- | --- | --- | --- | --- | --- | --- | --- | --- | --- | --- | --- |
| Control | Called cell^a)^ | 19981 | 83 | 0.415 | 85 | 4.25 | 1.02 | 76 | 0.380 | 86 | 4.30 | 1.13 |
| Control | background barcode^b)^ | 1472575 | 741 | 0.050 | 766 | 0.52 | 1.03 | 581 | 0.039 | 621 | 0.42 | 1.07 |
| KP_S | Called cell | 16580 | 509 | 3.070 | 2689 | 162.18 | 5.28 | 291 | 1.755 | 827 | 49.88 | 2.84 |
| KP_S | background barcode | 1461470 | 3649 | 0.250 | 4517 | 3.09 | 1.24 | 1701 | 0.116 | 1871 | 1.28 | 1.10 |
| PA_S | Called cell | 18436 | 91 | 0.494 | 99 | 5.37 | 1.09 | 2884 | 15.643 | 11704 | 634.84 | 4.06 |
| PA_S | background barcode | 803686 | 444 | 0.055 | 464 | 0.58 | 1.05 | 14000 | 1.742 | 23007 | 28.63 | 1.64 |

^a)^ “Called cell” denotes barcodes classified as cells by the cell-calling pipeline. ^b)^ “Background barcode” denotes observed raw barcodes not classified as cells, used to estimate background signal recovery; these are not experimentally validated empty microwells. ^c)^ Barcode classes and counts were defined at the cell-calling stage, before additional host-cell quality filtering. The larger background-barcode count reflects the raw barcode space before cell calling. ^d)^ KP- or PA-positive barcodes contain ≥1 corresponding bacterial 16S-derived UMI passing read-support and quality filters; positive rates use all barcodes in the respective class as the denominator. ^e)^ UMI density denotes KP- or PA-aligned bacterial UMIs per 1,000 barcodes in the respective class. Control, KP_S, and PA_S denote uninfected control, KP septic infection, and PA septic infection datasets, respectively. All metrics are descriptive technical QC measures, not biological replicates for statistical inference.

## Table S2. Bacterial target-reference alignment metrics for Klebsiella pneumoniae NTUH-K2044 and Pseudomonas aeruginosa PA14.

| Alignment metric | K. pneumoniae NTUH-K2044 | P. aeruginosa PA14 |
| --- | --- | --- |
| Uniquely mapped reads (n) | 616,964 | 3,213,974 |
| Multi-mapped reads (n) | 50,200 | 36,960 |
| Unique fraction among target-mapped reads (%)^a)^ | 92.48 | 98.86 |

^a)^ The unique fraction among target-mapped reads was calculated as uniquely mapped reads/(uniquely mapped reads + multi-mapped reads) × 100. Metrics were obtained from separate KP and PA reference alignments before feature assignment and downstream host-cell quality filtering; they describe within-reference alignment uniqueness, not cross-species classification accuracy. Bacterial 16S reads were aligned using STAR to the predefined K. pneumoniae NTUH-K2044 and P. aeruginosa PA14 reference sequences with a minimum matched-read requirement of 80 bases (--outFilterMatchNmin 80). Only uniquely mapped reads were assigned to bacterial 16S features for downstream analysis.

## Table S3. Primer sequence for RT-qPCR.

| Gene Name | species | Primer sequence_F | Primer sequence_R |
| --- | --- | --- | --- |
| Ifnb1 | Mouse | F:CAGCTCCAAGAAAGGACGAAC; | R:GGCAGTGTAACTCTTCTGCAT; |
| Il6 | Mouse | F:TAGTCCTTCCTACCCCAATTTCC; | R:TTGGTCCTTAGCCACTCCTTC; |
| Il1b | Mouse | F:GCAACTGTTCCTGAACTCAACT; | R:ATCTTTTGGGGTCCGTCAACT; |
| Cd38 | Mouse | F:TCTCTAGGAAAGCCCAGATCG; | R:GTCCACACCAGGAGTGAGC; |
| Cox1 | Mouse | F:TCAACATGAAACCCCCAGCCA; | R:GCGGCTAGCACTGGTAGTGA; |
| Cox2 | Mouse | F:AGACGAAATCAACAACCCCG; | R:GGCAGAACGACTCGGTTATCA; |
| Cox3 | Mouse | F:TGCAGGATTCTTCTGAGCGT; | R:AGTAGTGGGACTTCTAGAGGGTT; |
| Actb | Mouse | F:CAGATGCCACTACAGCACG; | R:CCTGCCGCTGCCATAGAAG; |
| CD38 | Human | F:CAACTCTGTCTTGGCGTCAGT; | R:CCCATACACTTTGGCAGTCTACA; |
| IL6 | Human | F:ACTCACCTCTTCAGAACGAATTG; | R:CCATCTTTGGAAGGTTCAGGTTG; |
| IFNB1 | Human | F:ATGACCAACAAGTGTCTCCTCC; | R:GGAATCCAAGCAAGTTGTAGCTC; |
| ACTB | Human | F:CATGTACGTTGCTATCCAGGC; | R:CTCCTTAATGTCACGCACGAT; |

## Table S4. Critical reagent utilized in this study.

| Reagent | Source | Identifier |
| --- | --- | --- |
| Rabbit Anti-Klebsiella Polyclonal Antibody, Unconjugated | Abcam | Cat#ab20947; RRID: AB_445933 |
| Mouse anti-CD38 monoclonal antibody | Proteintech | Cat#60006-1-Ig; RRID: AB_716994 |
| Rat anti-mouse CD38 monoclonal antibody | eBioscience | Cat#14-0381-82 |
| KP 16S-FISH | TinyGen | Cat#GW16S01 |
| FocuSCOPE 16S single-cell host–bacterial co-sequencing workflow | Singleron Biotechnologies | NA |
| SCOPE-chip single-cell capture system | Singleron Biotechnologies | NA |
| Mitochondrial Superoxide Assay Kit with MitoSOX Red | Beyotime | Cat#S0061M |
| Mitochondrial membrane potential assay kit with JC-1 | Beyotime | Cat#C2006 |
| Mito-Tracker Red CMXRos | Beyotime | Cat#C1049 |
| Lyso-Tracker Green, lysosomal green fluorescent probe | Beyotime | Cat#C1047S |
| LysoSensor Yellow/Blue DND-160 | Thermo Fisher Scientific / Invitrogen | Cat#L7545 |
| NAD⁺/NADH Assay Kit (WST-8) | MedChemExpress | Cat#HY-K0313 |
